# Dynamic graph convolution with comprehensive pruning and GNN classification for precise lymph node metastasis detection

**DOI:** 10.1038/s41598-026-37193-8

**Published:** 2026-01-30

**Authors:** Chaitra H. N., Shwetha N., Adarsh Rag S., Chandra Singh, Rangaswamy Y.

**Affiliations:** 1 Department of Computer Science and Engineering, Don Bosco Institute of Technology, Bangalore, India; 2 Department of Electronics and Communication, Dr. Ambedkar Institute of Technology, Bangalore, India; 3 Manipal Institute of Technology, Manipal Academy of Higher Education, Manipal, India; 4 Department of Electronics and Communication Engineering, Nitte (Deemed to be University), NMAM Institute of Technology Nitte, Mangalore, India

**Keywords:** Pruning, Segmentation, Embedding, Dynamic graph convolution, Lymph node, Cancer, Computational biology and bioinformatics, Mathematics and computing

## Abstract

Early and accurate detection of lymph node metastases is crucial for improving breast cancer patient outcomes. However, current clinical practices, including CT, PET imaging, and microscopic examination, are time-consuming and prone to errors due to low tissue contrast, varying lymph node sizes, and complex workflows. To address the limitations of existing approaches in lymph node segmentation, feature embedding, and classification, this study proposes a novel framework Graph-Pruned Lymph Node Detection Framework (GPLN-DF) that integrates a Dynamic Graph Convolution (DGC) autoencoder with Node Attribute-wise Attention (NodeAttri-Attention) for accurate lymph node segmentation. This segmentation is further refined using Comprehensive Graph Gradual Pruning (CGP) to reduce unnecessary parameters and computational costs. After segmentation, Hessian-based Locally Linear Embedding (HLLE) is applied for effective feature extraction and dimensionality reduction, preserving the geometric structure of lymph node regions. Finally, a Graph Neural Network (GNN) classifier enhanced with CGP is used to classify the segmented lymph nodes as metastatic or non-metastatic based on the extracted features. This comprehensive framework addresses challenges such as small lymph node size, shape variability, low contrast in medical imaging, and high computational burden. The model was evaluated on the CAMELYON17 dataset, achieving a classification accuracy of 98.65%, surpassing existing models in segmentation precision and classification performance.

## Introduction

Lymph Nodes play a crucial role in the immune system because it has the ability to filter fluids and capture viruses and bacteria. Any abnormalities in the lymph nodes are helpful for diagnosing diseases, since they frequently indicate pathological variations in their attributive zones. Evaluating the status of Auxiliary Lymph Nodes (ALNs) in breast cancer patients provides reliable and helpful information for both postsurgical and surgical care by identifying whether it is non-metastatic or metastatic^1^. The survival rate of breast cancer has improved through intervention and detection in early stages, but metastasis prevention and treatment have still not reached the clinical needs^2^. In histopathology, computer screens and digitized tissue slides have gradually replaced the traditional light microscope. Tissue sections are digitized to create Whole-Slide Images (WSIs), which are highly valuable for Machine Learning (ML) and Artificial Intelligence (AI) applications. This study is developed and evaluated exclusively on H&E-stained histopathology WSIs from the CAMELYON17 dataset, where accurate identification of lymph node metastasis is clinically critical. AI has effectively detected and graded numerous cancers, including prostate, breast, and colorectal cancer^3^. Unlike radiological modalities such as CT, MRI, or PET which are discussed only in related work or as potential extensions this work focuses entirely on WSI-based digital pathology, where challenges include extreme resolution, staining variability, small metastatic regions, and complex tissue heterogeneity.

Recently, automatic image segmentation models trained on large-scale datasets with carefully curated Ground Truth (GT) annotations provided by expert pathologists have achieved competitive results. In this context, GT refers to pixel-level annotations that serve as the gold standard for training and evaluation, rather than detection outputs. However, it remains difficult to achieve precise segmentation of lymph nodes from medical images due to their small size, low contrast, and heterogeneous appearance^4^. Significant advancement in automatic segmentation has been indicated by the development of ML and deep learning technologies, which offer effective solutions to address the drawbacks of manual segmentation. Even though, the automatic segmentation of metastatic lymph nodes was not properly described^5^. Therefore, a wide range of 2D approaches for the segmentation and detection of lymph nodes has been developed by various researchers. These methods use unidimensional measurements for determining the target lesions’ Short-Axis Diameter (SAD). Furthermore, when considering enlarged lymph nodes, 2D unidimensional method may underestimate both the growth and size of lymph nodes^6^. This diagnostic process is tedious and time-consuming. Most significantly, small metastases can be missed because they are so hard to detect. The low contrast and similar structures between metastasised regions and different adjacent tissues or organs make it extremely difficult to automatically detect the regional metastases, particularly from the WSIs^7^.

Recently, weakly supervised learning paradigms have gained attention in digital pathology as a means to reduce the heavy burden of dense pixel-level annotations. Fan et al.^8^ proposed PathMamba, a weakly supervised state space model for multi-class pathology image segmentation that leverages image-level labels and contrastive state space modeling to capture both fine-grained details and global contextual semantics. PathMamba demonstrates strong segmentation performance under limited supervision and achieves regionally consistent results on large pathology images. However, it does not explicitly model graph-based spatial relationships between tissue regions, nor does it address structural redundancy and computational efficiency, which are critical challenges for large-scale lymph node metastasis analysis in clinical settings.

While advances in segmentation and weak supervision improve detection accuracy, the growing scale and complexity of WSI-based models also necessitate efficient and compact architectures that can operate under strict computational constraints. In this context, deep neural network compression has become essential for modern lymph node segmentation and classification systems. Among various compression techniques, pruning has proven to be particularly effective in reducing model size and accelerating inference by eliminating redundant parameters. Structural pruning is especially advantageous, as it reduces computational cost and memory usage without requiring specialized hardware or software accelerators^9^. However, many existing pruning approaches rely on handcrafted rules defined by experts, which are time-consuming and may not yield optimally compressed models^10^. Lottery Ticket Hypothesis (LTH)-based pruning techniques primarily focus on pruning at initialization and provide limited insights into pruning during or after training^11^. Moreover, channel, filter, and parameter pruning methods may lead to irregular memory access patterns, necessitating specialized hardware support and potentially increasing computational overhead^12^. Passive filter pruning methods often estimate filter importance solely based on coefficient magnitudes, without considering their actual contribution to feature generation, which can degrade network performance by retaining weakly informative filters^13^.

In parallel, nonlinear dimensionality reduction approaches like, LLE aim to preserve the linear neighborhood structure present in the high-dimensional space while uncovering a low-dimensional embedding manifold. However, LLE’s effectiveness is greatly reduced when data are sparsely distorted or sampled by noise. Methods like, a Modified LLE (MLLE), the Robust LLE (RLLE), Local Tangent Space Alignment (LTSA), a RLLE utilising penalty functions, Stochastic Neighbor Embedding (SNE), Locality Preserving Projections (LPP), and Neighborhood Preserving Embedding (NPE) are used for addressing these issues. Additional works include the bearing feature extraction using Multi-Structure LLE (MS-LLE), an unsupervised adaptive embedding for dimensionality reduction, a Graph Regularized LLE (GLLE), and an unsupervised linear dimension reduction process that preserves local and global clustering structure^14^. The manifold-based techniques like LLE, laplacianeigenmaps, and their expanded algorithms like local preserving embedding and neighbor preserving embedding and local preserving projection can indicate the data’s intrinsic geometry structures, which have been broadly utilised in the HIS processing field. The main goal of the Graph Embedding (GE) model is to combine these existing approaches in order to get better understanding and to analyse these dimensionality reduction algorithms. However, GE techniques often rely on the Euclidean distance to investigate the intrinsic geometric relationship of data with a fixed neighborhood size, which is very sensitive to noise, reducing the discriminant performance of embedding features and affecting the accuracy of graph formation^15^.

Motivated by these limitations, the novelty of this work lies in the unified integration of graph-based segmentation, pruning, embedding, and classification into a single end-to-end framework tailored for lymph node metastasis analysis. Unlike existing approaches that treat segmentation, compression, and classification as independent stages, the proposed method introduces a tightly coupled pipeline where graph structures are dynamically optimized and reused across all stages. Specifically, this study is among the first to incorporate Comprehensive Graph Gradual Pruning (CGP) not only as a model compression strategy, but also as a structural refinement mechanism that enhances graph quality for both feature learning and classification. Furthermore, the combination of Dynamic Graph Convolution with Node Attribute-wise Attention enables precise segmentation of small and irregular lymph nodes, while Hessian-based Locally Linear Embedding (HLLE) preserves intrinsic manifold geometry prior to graph-based classification. This synergistic design significantly improves computational efficiency, interpretability, and diagnostic accuracy compared to conventional CNN-based or weakly supervised methods.

To address the limitations of existing approaches in segmentation, pruning, embedding, and classification, this study proposes a comprehensive framework, the Graph-Pruned Lymph Node Detection Framework (GPLN-DF) for lymph node metastasis detection and classification. The method integrates a Dynamic Graph Convolutional (DGC) autoencoder with Node Attribute-wise Attention (NodeAttri-Attention) for precise lymph node segmentation. To enhance computational efficiency, a Comprehensive Graph Gradual Pruning (CGP) technique is applied to reduce unnecessary parameters, prune weak graph structures, and make the model lightweight and faster. Following segmentation, Hessian-based Locally Linear Embedding (HLLE) is used for effective dimensionality reduction, preserving the manifold geometry and enhancing the quality of feature representation for classification tasks. After obtaining the lower-dimensional, high-quality feature set, a Graph Neural Network (GNN) integrated with CGP classifier is employed to analyze the segmented lymph nodes and classify them as metastatic or non-metastatic.

The main contributions of this article are,

- A novel GPLN-DF framework integrating DGC autoencoder with NodeAttri-Attention for accurate lymph node segmentation under challenging histopathological conditions.
- A comprehensive graph-aware pruning strategy (CGP) that jointly sparsifies weights, edges, and node features, fundamentally differing from prior pruning approaches that operate on a single structural level.
- HLLE-based dimensionality reduction that preserves intrinsic manifold geometry and synergizes with graph pruning to enhance learning stability and generalization.
- A CGP-integrated GNN classifier that achieves robust metastatic versus non-metastatic discrimination on the CAMELYON17 dataset.

The rest of the article is structured as follows: The Sect. Literature review represents the detailed literature which gives a short overview of certain papers that utilized different steps including pre-processing, segmentation and pruning. Section Proposed methodology demonstrates the proposed method. The result and discussion of this article is described in Sect. Implementation details, which includes implementation details, dataset description and comparative analysis. Finally, in Sect. Conclusion, the article is concluded with its major findings.

## Literature review

In this chapter, several articles have been reviewed based on the lymph node segmentation, in which various procedures are applied, including pre-processing, pruning and embedding. Therefore, the various existing approaches that are applied for all these steps are briefly specified here.

### Segmentation

In recent years, deep learning techniques for automatic segmentation of medical image have greatly developed. Here, SketchGNN, a convolutional GNN for semantic segmentation was introduced by Yang et al. (2021)^16^. SketchGNN extracts features at three levels using a static-dynamic branching network model and graph convolution to predict the per-node labels. However, this representation only captures features including proximity and node position, while lacking high-level semantic information, which limits the effectiveness of the current graph representation. Also, the learned class probability map ignores the relationship between elements. To address the limitation of missing global and local contextual dependencies, Agarwal et al. (2024)^17^ proposed a multi-scale dual-channel feature embedding decoder using parallel CNN encoders and attention-gated Swin Transformer blocks. Their approach effectively captures both short- and long-range dependencies while maintaining a manageable computational cost. However, transformer-based decoders still require large-scale datasets for stable training and are constrained by quadratic attention complexity, limiting scalability for high-resolution whole-slide images. To address this issue, Xu et al. (2022)^1^ proposed a framework difficulty-aware bi-network using a spatial attention constrained graph. This approach adaptively segments the image using various branches according to the difficulty grade of images. Additionally, it cannot be directly utilized for the 3D image segmentation results, such as CT and MRI, which limits the performance improvement. Additionally, this limits the model performance by restricting access to information outside the patches. To overcome this limitation, Su et al. (2024)^18^ proposed a GCUNet, a GNN-based contextual learning network for TLS semantic segmentation. Initially, it gradually aggregates fine-grained and long-range context outside the target to segment the given image patch. The segmentation mask is then predicted by integrating the detail and context of the target using a Detail and Context Fusion block (DCFusion). However, the diagnosis of cancer metastasis in lymph nodes takes more time and can sometimes lead to missed diagnoses. In parallel, Liu et al. (2021)^19^ explored deep learning–based prediction of axillary lymph node metastasis using contrast-enhanced CT images. Their work demonstrates the feasibility of learning discriminative features for lymph node assessment from imaging data; however, it focuses on classification rather than fine-grained segmentation and does not explicitly model spatial or topological relationships among tissue regions. In order to understand cancer metastasis in lymph nodes, Jayapal et al. (2025)^3^ proposed a framework that combines a GNN with DeepLab to effectively segment each location. To segment the lymph node regions from high-resolution PET and MRI scans, the proposed model combines DeepLab, a highly advanced convolutional neural network for image segmentation. Furthermore, this work did not include an optimization for computational efficiency and multi-institutional datasets, which limits the improvement in real-time processing and generalizability. It also did not focus on refining these approaches and applying them to other cancer kinds and diagnostic methods. More recently, transformer-based hybrid architectures have been explored for medical image segmentation. Sharma et al. (2025)^20^ proposed U-SwinTrans, which integrates Swin Transformer blocks into a U-shaped CNN architecture for improved global feature modeling. Although effective for skin lesion segmentation, such transformer-heavy models remain computationally expensive and have not been extensively validated for lymph node metastasis segmentation in whole-slide pathology images.

### Pruning

GNNs have recently attained great success in graph representation learning tasks. Chen et al. (2021)^21^ suggested Dynamic GNN (DyGNN), which speeds up GNNs by decreasing redundancies, enlightened by the fact that there are more message passing redundancies in GNNs. The architecture and algorithm co-design supports DyGNN. During execution, the suggested algorithm can dynamically prune edges and vertices without loss of accuracy. This dynamic pruning is supported by architecture and transforms it into performance enhancement. By pruning vertices and edges, DyGNN creates a new ways for accelerating GNNs in 2× speedup with 4% improved accuracy. However, this approach was not suitable for semantic segmentation networks. To overcome this issue, He et al. (2021)^22^ presented a Context-Aware Pruning (CAP) method emphasizes the importance of contextual information during channel pruning. Specifically, the embedded contextual information is formulated by using layer-wise channels association through the Context-Aware Guiding Module (CAGM). Conversely, this approach was inefficient because it did not examined from the approach of network model topology, and it was not suitable for all datasets. To overcome this problem, the neural network’s graph structure is examined, and Chen et al. (2023)^23^ proposed a Regular Graph Pruning (RGP) framework to achieve the one-shot neural network pruning. Here, when the network parameters are reduced highly, RGP shows stronger precision retention ability. The optimal edge distribution is obtained by swapping edges, which reduces the graph’s Average Shortest Path-Length (ASPL). Moreover, inference and training for GNN under tight latency constraints has become challenging as real-world input graphs continue to grow. Also, the structured pruning methods for GNNs presents performance scalability issues because the low-dimensional mapping of the pruned model cannot fully utilize the GPU’s vectorized hardware’s parallelism potential. Thus for resolving this issue, Gurevin et al. (2024)^24^ suggested PruneGNN, an optimized algorithm-architecture framework. PruneGNN achieves 2× and 4.6× speedup over baseline structured pruning and irregular pruning, respectively.

### Embedding

Numerous biomedical studies depend on the automatic segmentation and detection of biological objects in 2D and 3D image data. Spatial embedding-based instance segmentation techniques have demonstrated high-quality results in the natural image domain; however, their applicability to biomedical data remains largely unexplored. To address this issue, Lalit et al. (2022)^25^ proposed EMBEDSEG, which is an embedding-based segmentation approach aimed to segment instances of preferred objects that are present in 3D or 2D biomedical image data. To improve well logging data processing, Sun et al. (2024)^26^ presented a method using LLE for reducing dimensionality. LLE significantly decreases data dimensionality by preserving crucial local structure in a low-dimensional space and recognizing local linear associations, which is particularly useful for log data that frequently contains formation-specific information, like fluid content. This technique combines LLE with DGCN-TSA, has shown great accuracy in applications like Tarim Oilfield logging data analysis. But, it has been observed that this method did not sufficiently maintain the local topological structure of the original graphs, especially when nearby nodes belong to different categories. To address this issue, Liu et al. (2024)^27^ proposed a novel node embedding method called Locally Linear Contrastive Embedding (LLaCE). By employing locally linear formulation, LLaCE aims to maintain the graph data’s intrinsic geometric structure, ensuring that the local embedding space accurately reflects the local topological features. However, this method did not explore the alternative optimization methods or a general framework for local and global topology preservation, which limits its computational efficiency on large-scale networks.

literature review reveals that existing methods suffer from a lack of high-level semantic information, high time-consuming for diagnosing and segmentation, produce noise, face performance scalability issues, high computational costs, and may miss detections due to the small size of lymph nodes, which affects the segmentation accuracy and robustness. To solve these issues, a DGC autoencoder with NodeAttri-Attention integrated with CGP and HLLE is proposed. Tables 1 and 2 shows the research gap of the existing methods and comparison with prior approaches respectively.

**Table 1:** Research gap of the existing methods.

Author	Methodology	Objective	Key Innovations	Limitations	Applications
Yang et al. (2021)^16^	SketchGNN (Convolutional Graph Neural Network)	Semantic segmentation via GNN using static-dynamic branching and graph conv.	It extracts Multi-level features effective per-node prediction	Lacks high-level semantic information; ignores inter-element relationships	Semantic segmentation
Xu et al. (2022)^1^	Difficulty-aware Bi-network with spatial attention constrained graph	Adaptive image segmentation based on difficulty levels	Effective spatial attention; adaptive segmentation branches	It does not applicable to 3D segmentation (e.g., CT, MRI); limits patch info utilization	Medical image segmentation
Su et al. (2024)^18^	GCUNet -based contextual learning network with DCFusion	It helps to improve TLS segmentation via contextual learning and detail-context fusion	Aggregates fine-grained & long-range context; effective segmentation masks	Slow diagnosis for lymph node metastasis; may cause missed diagnoses	TLS semantic segmentation
Jayapal et al. (2025)^5^	GNN + DeepLab framework	It Segments lymph node regions in PET and MRI	High-res segmentation; combines GNN and DeepLab	Lacks real-time optimization; limited generalizability across institutions and cancer types	Lymph node cancer detection
Chen et al. (2021)^21^	DyGNN (Dynamic Graph Neural Network)	Speed up GNNs by pruning redundant message passing	Achieved 2× speedup with 4% better accuracy	Not suited for semantic segmentation networks	Graph acceleration
He et al. (2021)^22^	Context-Aware Pruning (CAP) with CAGM	It Improves the pruning using contextual information	Layer-wise context retained; guided channel pruning	Ineffective across different topologies and datasets	GNN compression and acceleration
Chen et al. (2023)^23^	Regular Graph Pruning (RGP)	One-shot neural network pruning with minimal accuracy loss	Maintains high precision; reduces ASPL by optimal edge swapping	Low-dimensional mapping hinders GPU optimization; latency issues in training/inference	Efficient GNN model compression
Gurevin et al. (2024)^24^	PruneGNN (Optimized architecture + algorithm)	Resolve latency and parallelism issues in GNNs	Achieves 2× and 4.6× speedups over structured and irregular pruning	Scalability challenges with increasing real-world graph size.	Real-time GNN for large-scale inputs
Lalit et al. (2022)^25^	EMBEDSEG (Embedding-based segmentation)	Instance segmentation for 2D/3D biomedical images	High-quality segmentation results in biomedical domain	Limited application beyond natural images; less tested in biomedical field	Biomedical object segmentation
Sun et al. (2024)^26^	LLE + DGCN-TSA	Improve well logging data dimensionality and structure preservation	Recognizes fluid content effectively; high accuracy in logging data	Fails to maintain local topology when nearby nodes differ in class	Oilfield log data analysis
Liu et al. (2024)^27^	LLaCE (Locally Linear Contrastive Embedding)	Preserve local geometric structure in embeddings	Accurate local topology reflection; strong preservation of local structure	No global framework; lacks optimization methods for large-scale graphs	Large-scale graph representation in biomedicine

**Table 2:** Comparison with prior approaches.

Dimension	CNN-based segmentation	Static graph CNNs	Attentionized GNNs	Pruning methods (CAP, RGP, DyGNN, PruneGNN)	Manifold methods (LLE, LLaCE)	Proposed DGC NAA + CGP + HLLE
Topology learning	None, grid only	Fixed adjacency	Usually fixed or lightly adaptive	Not a focus	Not a focus	Dynamic adjacency updated every encoding layer
Feature weighting inside a node	Implicit via filters	Equal across channels	Often node or edge weights only	Not applicable	Not applicable	Channel-wise Node Attribute attention that is context aware
Joint learning of features and graph	No	Partial	Partial	No	No	Yes, encoder learns features while topology evolves
Small, irregular metastases	Often missed	Sensitive to initial graph	Better than CNN but channel bias remains	Not targeted	Not targeted	Enhanced via attribute attention and evolving edges
Robustness to noise and staining variability	Moderately robust with augmentation	Sensitive to graph init	Moderate	Improves speed and size rather than robustness	Sensitive to noise	Pre-processing + dynamic graphs + HLLE crvature preservation
Model efficiency	Good to moderate	Moderate	Moderate	Strong in isolation	Not applicable	End-to-end CGP on weights, edges and attributes reduces compute while preserving accuracy
Interpretability	Limited	Low	Medium	Not applicable	Low	Attention weights expose clinically relevant channels and regions
End-to-end path to classification	Yes	Sometimes	Yes	Outside the main task	Outside the main task	Yes, segmentation → HLLE → GNN with CGP

## Proposed methodology

**Fig. 1:**
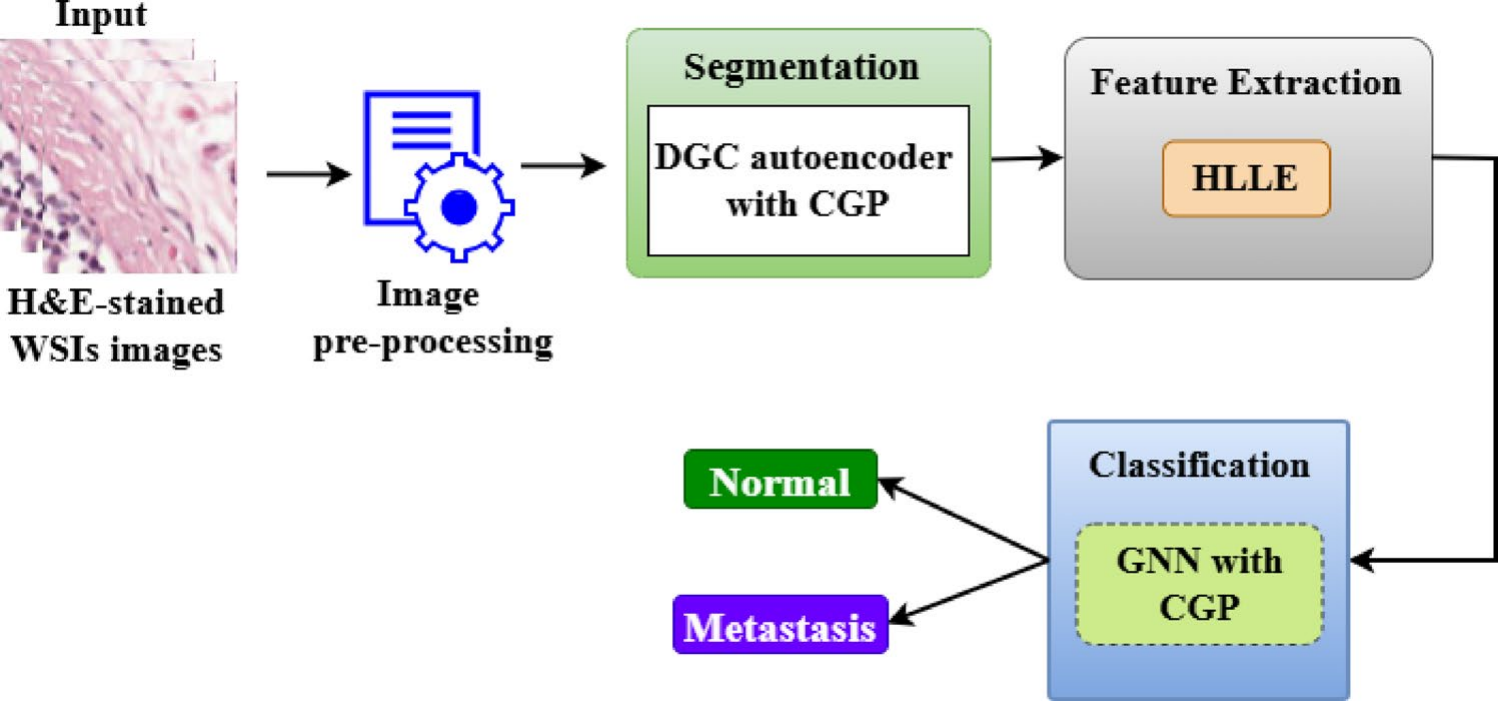
Block diagram for proposed Graph-Pruned Lymph Node Metastasis Detection Framework (GPLN-DF).

Figure 1 illustrates the overall architecture of the proposed framework for cancer metastasis detection in lymph nodes, hereafter referred to as the Graph-Pruned Lymph Node Detection Framework (GPLN-DF). The framework integrates multiple components into a unified end-to-end system. It begins with WSIs acquisition, where H&E-stained whole-slide images are pre-processed through normalization, noise reduction, and data augmentation to enhance quality and consistency. Following this, the DGC-CGP module is employed for precise segmentation by constructing graph-based representations of histopathology image regions and progressively refining them through pruning. The segmented outputs are then subjected to feature extraction using HLLE, which reduces high-dimensional data while preserving essential geometric structures. The refined features are input into GNNs integrated with CGP, which dynamically prune irrelevant nodes and edges to optimize graph topology. These GNNs extract meaningful relational patterns from the lymph node data, and the classification layer analyzes the learned features to predict whether a lymph node is metastatic or normal. Finally, the GPLN-DF provides a comprehensive end-to-end solution that enhances clinical decision-making by delivering accurate segmentation, computationally efficient feature reduction, and highly reliable classification of lymph nodes into metastatic or non-metastatic categories, with visualization support for interpretability.

The choice of graph-based modeling is motivated by the intrinsic irregularity and non-Euclidean nature of lymph node anatomy. Unlike grid-based CNNs, graph representations allow flexible modeling of heterogeneous tissue regions, variable shapes, and long-range contextual interactions. By treating superpixels or regions as nodes and their spatial or semantic relationships as edges, the framework captures clinically relevant structural dependencies that are difficult to represent using standard convolutional kernels. This graph-centric design forms the foundation for all subsequent stages in GPLN-DF, including pruning, embedding, and classification.

### Pre-Processing

**Fig. 2:**
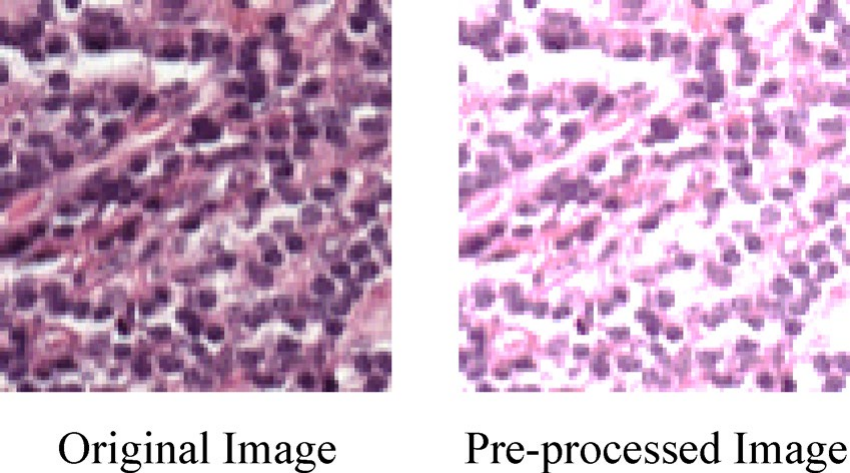
Visual representation of Pre-processed Image.

The intention behind the pre-processing in DGC autoencoder with NodeAttri-Attention for evaluating cancer metastasis in lymph nodes is to improve H&E-stained WSI quality, thereby enhancing the segmentation accuracy. This contains many significant processes, such as noise reduction, augmentation and image normalization. To ensure consistent input across dissimilar images, image normalization plays a crucial role in standardizing pixel intensity values. This is achieved by normalizing pixel values to a range between $\:0$ and$\:\:1$. To develop the clarity of lymph node structures and to reduce artifacts, noise reduction methods including Gaussian filtering or median filtering is applied. Lastly, data augmentation methods like flipping, scaling and rotation are employed to artificially develop the diversity of the training datasets and improve the resistance of model to changes in the input images. In this study, each original image was augmented into 5 additional variants (rotation, horizontal/vertical flipping, and two scaling factors), producing a total of approximately $\:{N}_{aug}$ images from the original $\:N\:$samples. This resulted in a five-fold increase in the effective training set size, which greatly reduced the risk of overfitting. This complete pre-processing pipeline helps to produce high-quality input for this segmentation, which increase the accuracy of cancer metastasis detection and analysis^5^. Figure 2 shows the pre-processed image.

There are three distinct stages in pre-processing such as, noise reduction, normalization, and augmentation, which are signified as below:

The first stage of pre-processing becomes normalization, which is mathematically expressed in Eq. (1)

1 $$\:{I}_{norm}\left(x,\:y\right)=\frac{I\left(x,\:y\right)-\mu\:}{\sigma\:}$$

Where $\:I\left(x,\:y\right)\:$represents the original pixel intensity at spatial location $\:\left(x,\:y\right)$ in the input histopathology image. The parameter µ denotes the mean pixel intensity computed over the entire image, while σ corresponds to the standard deviation of pixel intensities, capturing the overall contrast distribution. The normalized pixel value $\:{I}_{norm}\left(x,\:y\right)\:$is obtained by standardizing the original intensity using these statistics, ensuring consistent intensity scaling across whole-slide images and improving training stability and convergence of the proposed model. The second stage of pre-processing is noise reduction that is expressed in Eq. (2)

2 $$\:{I}_{filtered}\left(x,\:y\right)=\frac{1}{{\mathrm{k}}^{2}}\sum\:_{\mathrm{l}=\frac{\mathrm{k}}{2}}^{\frac{\mathrm{k}}{2}}\sum\:_{\mathrm{m}=-\frac{\mathrm{k}}{2}}^{\frac{\mathrm{k}}{2}}\mathrm{I}\left(\mathrm{x}+\mathrm{l},\:\mathrm{y}+\mathrm{m}\right).\mathrm{W}\left(\mathrm{l},\:\mathrm{m}\right)$$

Where, the pixel value after filtering is denoted as$\:\:{I}_{filtered}\left(x,\:y\right)$, $\:W\left(l,\:m\right)$ signifies the filter weights. E.g., Gaussian kernel, and the filter size is represented as$\:\:k$.

The third stage of pre-processing becomes augmentation, data augmentation methods use operations including rotation$\:\:\theta\:$, flipping and scaling $\:s$ to transform the image$\:\:I\left(x,\:y\right)$. For instance,$\:{I}_{rot}\left(x,\:y\right)$ represents the rotated image and it can be computed as in Eq. (3):

3 $$\:{I}_{rot}\left(x,y\right)=I\left({x}^{{\prime\:}},{y}^{{\prime\:}}\right)$$

After the rotation transformation, the coordinates $\:{\mathrm{x}}^{{\prime\:}},{\mathrm{y}}^{{\prime\:}}$ are defined in Eq. (4):

4 $$\:\left[\genfrac{}{}{0pt}{}{{x}^{{\prime\:}}}{{y}^{{\prime\:}}}\right]=\left[\genfrac{}{}{0pt}{}{\mathrm{cos}\left(\theta\:\right)}{\mathrm{sin}\left(\theta\:\right)}\genfrac{}{}{0pt}{}{-\mathrm{sin}\left(\theta\:\right)}{\mathrm{cos}\left(\theta\:\right)}\right]\left[\genfrac{}{}{0pt}{}{x}{y}\right]$$

Here, θ represents the rotation angle and $\:(x{\prime\:},y{\prime\:})\:$transformed coordinates.

### Segmentation using DGC with CGP

Graph-based segmentation is particularly well suited for lymph node analysis due to the irregular morphology, heterogeneous texture distribution, and ambiguous boundaries of metastatic regions. Conventional CNN-based segmentation approaches assume fixed local neighborhoods and struggle to adapt to variable anatomical structures. In contrast, dynamic graph convolution enables the model to adaptively redefine neighborhood relationships based on learned feature similarity, allowing robust handling of size variation, shape irregularity, and partial-volume effects commonly observed in lymph node images.

In the proposed framework for lymph node metastasis assessment, the segmentation process is executed through a robust and adaptive method called DGC with CGP. This segmentation approach is designed to handle the complex and irregular structures inherent in medical images, particularly lymph node tissues exhibiting metastatic spread. The segmentation pipeline commences with data preprocessing, where lymph node images are normalized and transformed into feature representations suitable for graph-based processing. Each image is initially partitioned into superpixels or local regions, which serve as the nodes $\:\mathrm{V}$ of a constructed graph $\:\mathrm{G}=\left(\mathrm{V},\mathrm{E}\right)$. The edges $\:\mathrm{E}$ of this graph capture the neighborhood relationships among these nodes, either through spatial proximity or feature similarity. These relationships are encoded into an adjacency matrix $\:\mathrm{A}\in\:{\mathrm{R}}^{\mathrm{N}\times\:\mathrm{N}}$, where $\:\mathrm{N}$ denotes the number of nodes. The adjacency matrix evolves dynamically during training through learnable parameters that reflect the underlying data manifold.

#### Graph construction and node attributes

To generate graph nodes in a spatially meaningful and computationally efficient manner, each input lymph node image is first partitioned into superpixels using the Simple Linear Iterative Clustering (SLIC) algorithm. SLIC groups neighboring pixels into compact, approximately homogeneous regions based on color intensity and spatial proximity, which is particularly suitable for histopathological images where tissue structures exhibit local consistency. Each superpixel corresponds to one graph node, allowing the constructed graph to preserve anatomical boundaries while significantly reducing graph size compared to pixel-level representations.

Feature extraction for each node is performed using the output of the 3D encoder. Specifically, the encoder produces a volumetric feature map $\:F\in\:{\mathfrak{R}}^{H\times\:W\times\:D\times\:C}$, where $\:C$ denotes the number of feature channels. For a given superpixel region $\:{v}_{i}$, all voxel-level feature vectors within the region are aggregated using average pooling across the spatial dimensions, yielding a fixed-length descriptor $\:{X}_{i}\in\:{\mathfrak{R}}^{C}$. This aggregation captures the dominant appearance, texture, and contextual cues of the region while suppressing noise from individual pixels. By stacking all node feature vectors, the node attribute matrix $\:X\in\:{\mathfrak{R}}^{{N}_{V}\times\:C}$ is obtained, where $\:{N}_{V}$​ denotes the total number of superpixels. This process establishes a direct and consistent mapping between the 3D encoder output and the graph-based representation used for dynamic graph convolution.

The initial adjacency matrix $\:{A}_{1}$ is computed using the $\:{L}_{1}$**-**distance similarity between node attributes as follows:

5 $$\:{\phi\:}_{ij}=exp\left\{-{\Vert{X}_{i}-{X}_{j}\Vert}_{1}\right\}$$

The similarities are rescaled to the range $\:\left[0,\:1\right]$ using the exponential operation exp. The edge weight of two nodes is higher (smaller) and nearer to 1 (0) if they are more similar. The adjacency matrix is gained by performing Laplace normalization on$\:{\Phi\:}=\left[{\phi\:}_{ij}\right]\in\:{\mathfrak{R}}^{{N}_{V}\times\:{N}_{V}}$.

6 $$\:{A}_{1}={D}_{1}^{-\frac{1}{2}}{\Phi\:}{D}_{1}^{-\frac{1}{2}}$$

Here, $\:{\left({D}_{1}\right)}_{ii}=\sum\:_{j}{{\Phi\:}}_{ij}$, and a diagonal matrix is denoted by $\:{D}_{1}$. The graph topology and edge connections are reflected in the adjacency matrix. Equation (6) initializes the adjacency matrix for this dynamic graph, and it is further developed and learned through DGC encoding layers during training.

A graph node’s attribute vector contains diverse attributes from different $\:F$ channels. The values of the node attribute vectors should contribute differently in weight to the node’s relation representation learning since various channels may have varying importance in decision-making. To signify the node attributes with attention weights, a novel NodeAttri-Attention technique is suggested.

#### NodeAttri-Attention mechanism

The NodeAttri-attention mechanism is defined as follows by considering contextual relations and channel-wise variations between the multiple nodes’ attribute vectors of various nodes: the attention weight vector of node $\:{v}_{i}$ is computed as follows, given$\:{\left({X}_{i}\right)}^{T}$, which represents the transposed $\:i$-th row of$\:\:X$:

7 $$\:{u}_{i}={H}_{a}\:tanh\left({W}_{a}{\left({X}_{i}\right)}^{T}+{b}_{a}\right)$$

Here, the attention weight matrix, which indicates the contextual associations among graph nodes, is denoted by$\:{H}_{a}$, the weight matrix is represented by $\:{W}_{a}$ and the bias vector is denoted by$\:{b}_{a}$. During the training,$\:{H}_{a}$,$\:{W}_{a}$ and $\:{b}_{a}$ are learned automatically and initialized randomly.

The following is the definition of normalized attention weight $\:{\alpha\:}_{it}$ of $\:{t}^{th}$ attribute of$\:{u}_{i}$:

8 $$\:{\alpha\:}_{it}=\frac{exp\left({u}_{it}\right)}{\sum\:_{t}exp\left({u}_{it}\right)},\:t=1,\dots\:,C$$

Equation (8) defines the normalized attention weight$\:\:{\alpha\:}_{it}$​, which can be interpreted as the signal amplification coefficient assigned to the $\:{t}^{th}$attribute of node $\:{u}_{i}$​. Here, $\:{u}_{i}\in\:{\mathfrak{R}}^{C}$ is a channel-wise attention score vector obtained from Eq. (7), and $\:{\alpha\:}_{it}$ ​ represents its$\:\:{t}^{th}$ component after softmax normalization. Thus, $\:{\alpha\:}_{i}=\left[{\alpha\:}_{i1},\dots\:,{\alpha\:}_{iC}\right]\in\:{\mathfrak{R}}^{C}$ is a channel-wise attention vector whose elements sum to one.

Consequently, the attention enhanced attribute vector $\:\stackrel{\sim}{{X}_{i}}$of$\:{X}_{i}$, node-attribute-wise, is:

9 $$\:\stackrel{\sim}{{X}_{i}}={\alpha\:}_{i}\otimes\:{X}_{i}+{X}_{i}$$

Equation (9) describes how the attention-enhanced attribute vector$\:\:\stackrel{\sim}{{X}_{i}}$ is constructed. The attention weight $\:{\alpha\:}_{i}$​ acts as a modulating signal, performing element-wise multiplication with the attribute vector $\:{X}_{i}$​. Both $\:{\alpha\:}_{i}$ ​ and $\:{X}_{i}$​ are C-dimensional vectors, and ⊗ denotes element-wise (Hadamard) multiplication, ensuring dimensional consistency. The residual connection preserves the original node attributes while adaptively amplifying informative channels and suppressing less relevant ones. This formulation improves robustness against noise and enhances discriminative feature learning at the node level.

#### DGC autoencoder

In conventional graph convolution, the adjacency matrix remains fixed throughout training. However, such static structures may fail to capture evolving relationships between nodes in complex medical images. To address this limitation, a Dynamic Graph Convolution (DGC) mechanism is employed, allowing the graph topology to be updated across encoding layers based on learned node representations.

##### DGC-Encoder

The first encoding layer explicitly operates on the attention-enhanced node attribute matrix $\:\stackrel{\sim}{X}\:$obtained from Eq. (9) and the initial adjacency matrix $\:{A}_{1}$​:

10 $$\:{Y}_{1}={f}_{LeakyReLU}\left({A}_{1}\stackrel{\sim}{X}{W}_{1}\right)$$

Where, weight matrix is represented by$\:{W}_{1}$, and $\:{f}_{LeakyReLU}$ denotes the Leaky ReLU. The first layer’s output feature matrix is denoted by$\:{Y}_{1}$, which consists of every node’s learned representative features. In graph convolution, the $\:i$-th column in $\:{W}_{1}$ is regarded as the $\:i$-th filtering window. $\:{W}_{1}$must be learned throughout the training process. By explicitly using the attention-enhanced attributes $\:\stackrel{\sim}{X}$, the encoder ensures that channel-wise importance learned by the NodeAttri-Attention mechanism directly influences graph convolution and downstream topology evolution. In subsequent encoding layers, the adjacency matrix is dynamically updated using the newly learned node representations, enabling adaptive modeling of complex and irregular lymph node structures.

##### Dynamic evolution of graph topology

Primarily, $\:{Y}_{k-1}$ is concatenated and the node attribute matrix $\:\stackrel{\sim}{X\:}$as $\:{\stackrel{\sim}{Y}}_{K-1}$, using the output feature map from the $\:\left(k-1\right)$-th graph convolution layer, represented by$\:{Y}_{k-1}$. Thus, during the dynamic evolution process of graph topology, both the new representative features of nodes and the original attribute details are considered. Specifically, the concatenation process is defined as,

$$\:{\stackrel{\sim}{Y}}_{k-1}=\left[{Y}_{k-1}\right|\left|\stackrel{\sim}{X\:}\right]$$

where $\:{Y}_{k-1}\in\:{\mathfrak{R}}^{N\times\:{d}_{k-1}}$​ denotes the output feature map of the $\:(k-1)$-th graph convolution layer, $\:\stackrel{\sim}{X\:}\in\:{\mathfrak{R}}^{N\times\:{d}_{0}}$ ​represents the original node attribute matrix derived from the encoder, and ∥ denotes channel-wise concatenation. As a result, each node embedding in$\:{\stackrel{\sim}{\:Y}}_{k-1}$ jointly encodes both the newly learned semantic representation and the initial low-level attribute information.

This hybrid representation is crucial for robust dynamic topology evolution. Using only $\:{Y}_{k-1}$​ to compute inter-node distances may cause progressive loss of fine-grained spatial and textural cues due to repeated aggregation, especially in deeper layers where oversmoothing can occur. By reintroducing the original node attributes at every layer, the model preserves stable anatomical and appearance-related information while still benefiting from hierarchical feature abstraction. This mixture enables the distance computation to remain sensitive to both evolving semantic similarity and intrinsic node characteristics, which is particularly important for distinguishing small metastatic regions that exhibit subtle texture variations similar to surrounding lymph tissue.

Consequently, the evolved adjacency matrix $\:{\stackrel{\sim}{A}}_{k}$​ reflects a dynamically adaptive graph structure that balances learned contextual relationships with original attribute consistency. This design ensures that topology updates are not purely driven by transient deep features but remain grounded in the initial image-derived properties, thereby enhancing structural fidelity, boundary awareness, and segmentation robustness throughout the encoding process.

The evolved adjacency matrix is represented by$\:\stackrel{\sim}{A}$, which is obtained as follows:

11 $$\:{\stackrel{\sim}{A}}_{k}={\stackrel{\sim}{D}}_{k}^{-\frac{1}{2}}{{\Phi\:}}_{k}{\stackrel{\sim}{D}}_{k}^{-\frac{1}{2}},\:{\stackrel{\sim}{{\Phi\:}}}_{{k}_{ij}}=exp\left\{-{\parallel\:{\stackrel{\sim}{Y}}_{k-1i}-{\stackrel{\sim}{Y}}_{k-1j}\parallel\:}_{1}\right\}$$

where, the $\:k$-th layer’s dynamic output is expressed as follows, and$\:{\left({\stackrel{\sim}{D}}_{k}\right)}_{ii}=\sum\:_{j}{\stackrel{\sim}{{\Phi\:}}}_{{k}_{ij}}$.

12 $$\:{Y}_{k}={f}_{LeakyReLU}\left({\stackrel{\sim}{A}}_{k}{Y}_{k-1}{W}_{k}\right),\:k=2,\dots\:,{L}_{e}$$

where$\:\:{L}_{e}$ denotes the number of encoding layers^6^.

#### Proposed DGC autoencoder with NodeAttri-Attention

The proposed framework combines the strengths of DGC and NodeAttri-Attention into a unified autoencoder architecture for precise lymph node segmentation. Traditional graph convolution methods rely on fixed adjacency matrices, which limits their ability to capture evolving structural patterns in medical images. In contrast, our DGC module dynamically updates the graph topology at each encoding layer by recalculating edge weights according to the evolving feature representations. This dynamic evolution allows the network to adaptively model the irregular shapes, heterogeneous textures, and complex spatial dependencies observed in lymph node tissues.

Furthermore, lymph node image regions (represented as graph nodes) contain multi-channel attributes such as intensity, texture, and edge descriptors. Conventional methods treat all attributes equally, which can dilute clinically significant features. To overcome this, the NodeAttri-Attention mechanism assigns context-aware importance weights to each channel of the attribute vector. For instance, channels that capture high-frequency edge information may receive higher weights in boundary regions, while intensity-related channels may dominate homogeneous regions. By integrating these adaptive weights into the graph convolution process, the model ensures that more discriminative features contribute strongly to segmentation accuracy.

Another major feature of the framework is its joint learning of feature representations and graph structure. While DGC captures evolving inter-node dependencies, NodeAttri-Attention refines node-level attributes, and together they produce embeddings that are both topologically and semantically optimized. This dual learning improves robustness against imaging noise, variations in resolution, and anatomical differences across patients. The entire framework operates in an autoencoder structure, where the encoder progressively refines graph embeddings through DGC with NodeAttri-Attention, while the decoder reconstructs the segmentation map. This ensures that the compressed embedding space preserves essential structural and clinical information for accurate lymph node metastasis detection.

### Pruning of DGC using CGP

**Fig. 3:**
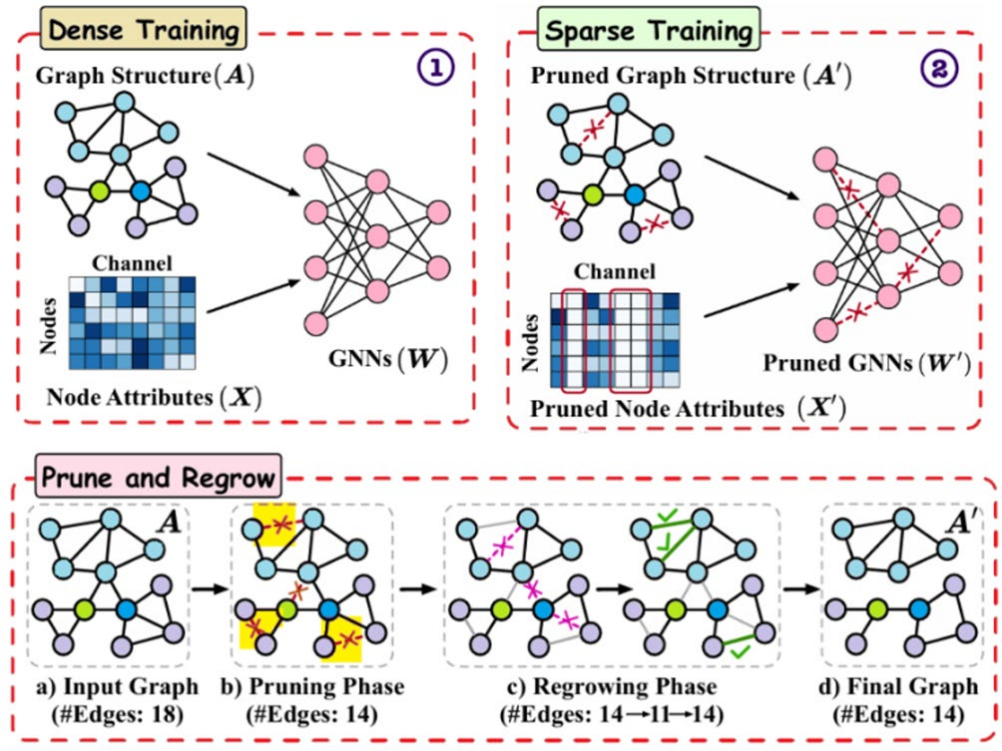
Architecture of CGP method.

In a single training process, the CGP can obtain DGC autoencoder with NodeAttri-Attention model weights and the sparse graphs $\:\left({\mathrm{A}}^{{\prime\:}},\:{\mathrm{X}}^{{\prime\:}}\right)$ that perform well. The complete sparsification technique is introduced, which co-sparsifies all three elements found in DGC techniques. The gradual magnitude pruning technique performing the sparsification prior to convergence during the training stage is described later. In order to improve the pruning process, the regrowth method is given at the end, which aids to recovering the pruned complex connections. The architecture of CGP method is displayed in Fig. 3.

In this study, the pruning rate refers to the overall sparsity applied jointly across three components of the DGC model: (i) model weights $\:\left(W\right)$, (ii) graph edges in the adjacency matrix $\:\left(A\right)$, and (iii) node feature dimensions $\:\left(X\right)$. For example, a 50% pruning rate means that half of the least important weights, edges, and feature dimensions are removed, while a 99% pruning rate retains only 1% of the original connections and features. This unified pruning strategy allows us to assess how performance changes as redundancy in all three components is gradually reduced.

The motivation for jointly pruning model weights, graph edges, and node features arises from their intrinsic coupling in graph convolution operations. In DGC layers, feature propagation is governed by the interaction$\:\:Z=AXW$, implying that redundancy in any one component inevitably induces redundancy in the others. Pruning node feature dimensions $\:X$ directly removes redundant input channels, which consequently eliminates unnecessary columns in the weight matrices $\:W$ that no longer contribute to feature transformation. This reduces weight redundancy at its source and simultaneously lowers the need for dense graph connectivity, as fewer informative features require propagation across edges. Conversely, pruning the graph structure $\:A$ constrains message passing to semantically meaningful neighborhoods, preventing feature oversmoothing and preserving node feature expressiveness. A sparser graph reduces the aggregation complexity, enabling the remaining weights to learn more stable and discriminative mappings with lower parameter redundancy.

By performing pruning jointly and gradually, CGP enables mutual reinforcement among these components: feature pruning simplifies weight learning, graph pruning refines feature aggregation, and weight pruning compresses the parameter space. The regrowth mechanism further ensures that informative features, edges, or weights that are mistakenly pruned can be recovered based on gradient feedback, maintaining model expressiveness while achieving high sparsity.

#### The model weight pruning (W)

In particular, a non-differentiable binary mask $\:{m}_{w}$ is generated during the model initialization stage, which is equal in size to the weight of model$\:\:W$. Initially, all the components in the mask are set to unity. This pruning approach sets the parameters below the threshold to zero, updates the mask matrix at regular intervals. The weight matrix is then multiplied by the updated mask to identify which weights are involved in the graph’s subsequent forward performance. This method can be explained as follows:

13 $$\:\begin{array}{c}idx=TopK\left(-\left|W\right|,\left[{p}_{w}{\Vert W\Vert}_{0}\right]\right);\\\:{m}_{w}^{{\prime\:}}=Zero\:\left({m}_{w},\:idx\right);\\\:{W}^{{\prime\:}}={m}_{w}^{{\prime\:}}\odot\:W,\end{array}$$

Where $\:Zero$ stands for the function, which sets the values in $\:{m}_{w}$ with indices $\:idx$ to$\:\:0$, $\:TopK$ denotes the function that yields the indices of $\:top\:\lceil{p}_{w}{\Vert W\Vert}_{0}\rceil$ values in$\:\left|W\right|$, where $\:{p}_{w}$ stands for the sparsity of model weights, that means, $\:1-{p}_{w}$ proportion of weights are retained for the following iteration, $\:{W}^{{\prime\:}}$ denotes the pruned weight matrix,$\:\lceil\bullet\:\rceil$ denotes the rounding up operator, $\:{\Vert W\Vert}_{0}$ represents the total number of model weights, and $\:\odot\:$ signifies the element-wise product. By default, global pruning is adopted, which states to pruning the weights across various layers together rather than individually.

#### The graph structure pruning (A)

The adjacency matrix is generally fixed with training process, in contrast to the model weights that are modified throughout each optimizer update step. Consequently, the adjacency attention mask $\:{m}_{a}\in\:{\mathfrak{R}}^{{\Vert A\Vert}_{0}}$ is introduced. This is a differentiable and sparse soft-mask, where $\:{\Vert A\Vert}_{0}$ denotes the total number of edges considered for pruning. The soft-mask$\:{m}_{a}$, which functions as edge-weight and is same as the attention values in Graph Attention Network (GAT), is sent to the DGC model after every optimizer update step. These masks are modified by fixing all parameters that fall below the threshold to zero during the regular intervals, generally in accordance with the pruning interval of model weight.

14 $$\:\begin{array}{c}idx=TopK\left(-\left|{m}_{a}\right|,\:\lceil{p}_{a}{\Vert A\Vert}_{0}\rceil\right);\\\:{m}_{a}^{{\prime\:}}=Zero\left({m}_{a},\:idx\right);\\\:{A}_{nonzero}^{{\prime\:}}={m}_{a}^{{\prime\:}}\odot\:{A}_{nonzero},\end{array}$$

Where $\:{p}_{a}$ denotes the graph structure sparsity, $\:{A}_{nonzero}$ represents the edge index^2^, $\:{A}_{nonzero}^{{\prime\:}}$ stands for pruned edge index employed in the following training iteration, and the indexing operation is denoted by$\:\:idx$.

#### The node feature pruning (X)

In addition to the pruning methods of weight and graph structure, the node features’ dimension is also pruned, which is the main element of DGC. While sparsifying weights removes the elements of the matrices, eliminating input features corresponds to eliminating columns or rows in the weight matrices. Furthermore, for downstream tasks like node classification, the node features usually have lot of redundancy. Consequently, the feature attention mask $\:{m}_{x}\in\:{\mathfrak{R}}^{d}$ is introduced, a differentiable and sparse soft-mask, where$\:\:d$ represents the node features’ dimension, similar to graph structure pruning. In order to direct the pruning of input node feature, this soft-mask is used on the input layer. Then the formulation becomes:

15 $$\:\begin{array}{c}idx=TopK\left(-\left|{m}_{x}\right|,\lceil{p}_{x}d\rceil\right);\\\:{m}_{x}^{{\prime\:}}=Zero\left({m}_{x},\:idx\right);\\\:{X}^{{\prime\:}}={m}_{x}^{{\prime\:}}\odot\:X,\end{array}$$

Where $\:{X}^{{\prime\:}}$ stands for pruned feature matrix, and$\:{p}_{x}$ for sparsity of feature dimension utilized in the following training iteration. Here, only the input features are pruned while channel pruning including embedding pruning is excluded.

#### Gradual magnitude pruning

A schedule is frequently used to train the candidate elements’ sparsity, as presented in the previous part. Three different classes of training schedules are identified by the prior works: train-then-sparsify, sparsify-during-training and before-training-sparsify. (1) The train-then-sparsify process executes sparsification after a procedure of typical dense training, which means it continues for $\:T\:$iterations until convergence, after a retraining procedure is performed. This usually requires to be executed over more cycles, resulting in extremely high computational cost. In addition to that, over-fitting may result from training a dense model to converge, which is difficult to fix with the ensuing pruning operation. (2) Sparsification is performed by the before-training-sparsify schedule prior to the primary training process, but it typically affects from insufficient performance. (3) By performing sparsification before the model’s convergence$\:\left(T\right)$, the sparsify-during-training schedule can exhibit both training/ inference efficiency and comparable performance simultaneously.

The train-then-sparsify schedule is used to train the LTH-based DGC sparsification approaches, but it leads to a significant training computation cost. So, sparsify-during-training schedules are used and applied to DGC for the first time generalization. Specifically, the model weights are gradually pruned in addition to the DGC specific elements like node features and graph structure, as in convolutional DNNs. In order to achieve the required sparsity, the elements are pruned over $\:n$ pruning iterations using the gradual magnitude pruning. At every $\:t$steps, sparsification of all elements is performed, then the pruning rate of each pruning iteration becomes

16 $$\:{p}_{t}={p}_{f}+\left({p}_{i}-{p}_{f}\right){\left(1-\frac{t-{t}_{0}}{n\varDelta\:t}\right)}^{3},t\in\:\left\{{t}_{0},{t}_{0}+\varDelta\:t,\dots\:,{t}_{0}+n\varDelta\:t\right\}$$

where $\:{p}_{i}$ denotes the initial degree of sparsity, $\:{t}_{0}$ is the gradual pruning’s starting epoch, $\:{p}_{f}$ denotes the target sparsity, and $\:{p}_{t}$ stands for $\:{p}_{x}$, $\:{p}_{w}$, or $\:{p}_{a}$. When there are many redundant connections in the first phase, the aforementioned gradual pruning method helps to a fast pruning. As there are lesser connections remain, the connections that are pruned all time gradually decreases. As this now the typical technique for pruning during training phase, the pruning rate is used to prune elements, which have the least magnitude, as shown in Eqs. (13), (14), and (15).

#### Regrowth

The significant information loss may arise due to premature pruning at the pruning process, particularly in the initial iterations. The regrowth schemes such as gradient based regrowth, random regrowth, and momentum based regrowth can be incorporated into the gradual pruning method to correct the incorrect pruning. Before performing regrowth, a proportion of elements is eliminated to make sure that the model maintains the original size.

As an example, the edges in graph can be regrown $\:\left(A\right)$ using a gradient-based regrowth technique. Since the lesser magnitude denotes that either the gradient of connection is high or small number of oscillations influenced the gradient direction, the insignificant associations as those with the lowest magnitude of edge weights $\:{m}_{a}$ is first identified. So, these edges can be removed because they have a lesser influence to the training loss reduction. The $\:r$-th proportion of insignificant edges with the smallest magnitude is first eliminated, considering$\:\:r$ is the regrowth ratio.

17 $$\:\genfrac{}{}{0pt}{}{idx=TopK\left(-\left|{m}_{a}\right|,\:r\right);}{{A}_{nonzero}^{{\prime\:}}=Zero\left({A}_{nonzero},\:idx\right),}$$

where, $\:{A}_{nonzero}$ represents the edge matrix following the removal of $\:r$-th proportion of edges. Next, new connections corresponding to the $\:r$-th proportion are regenerated in accordance with the gradient magnitude, which is specified by

18 $$\:\genfrac{}{}{0pt}{}{idx=TopK\left(\left|{g}_{i\notin\:{A}_{nonzero}^{{\prime\:}}}\right|,\:r\right);}{{A}_{nonzero}^{"}={A}_{nonzero}^{{\prime\:}}+Zero\left({A}_{nonzero,\:}idx\right),}$$

Where $\:\left|{g}_{i\notin\:{A}_{nonzero}^{{\prime\:}}}\right|$ denotes the momentum or gradient magnitude of the eliminated edges, and $\:{A}_{nonzero}$ represents the edge matrix after the entire regrowth procedure. The regrowth of $\:A$ is similar to that of other two elements, $\:W$and $\:X$. From the starting of training to the ending of pruning, the above mentioned regrowth process is performed at every $\:t$ intervals. Based on the analysis of previous works, the regrowth method is significantly high training efficient.

**Algorithm 1:**
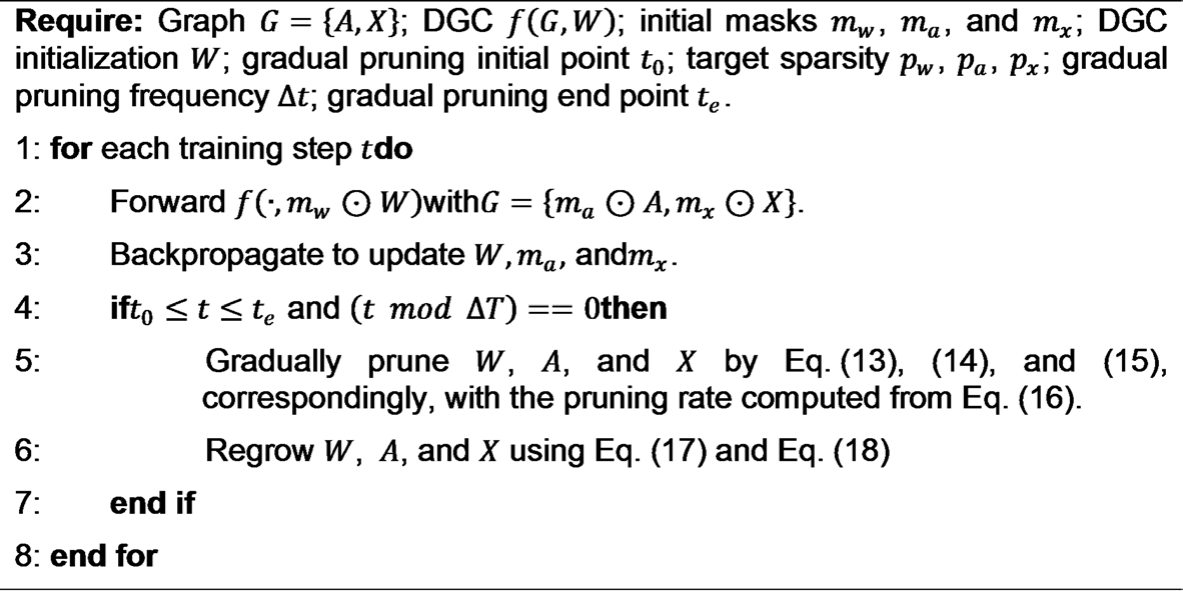
The pseudocode for the introduced CGP method.

Algorithm 1 shows the proposed CGP. It is used to enhance the efficiency of DGC model by pruning parameters. Primarily, it prune the three parameters: the adjacency matrix $\:A$, the model weights $\:W$, and the node features $\:X$. During the each step of training, t performs forward using graph data, followed by backpropagation to update the parameters. Between $\:{t}_{0}$to $\:{t}_{e}$, it prunes $\:W$, $\:A$ and $\:X$ based on pruning rates. Then regrowth is incorporated to restore the previously pruned elements. This pruning regrowth method helps to preserve lymph node segmentation accuracy, and it is helpful for complex graph structures.

The complete DGC-CGP architecture integrates DGCs with pruning and regrowth cycles. The forward operation at any point can be expressed as:

19 $$\:Z=f\left(A,\:X,\:W\right)$$

Throughout training, pruning reduces redundancy in $\:A,\:X,\:W$ for computational efficiency, while regrowth ensures the recovery of crucial structures. This dual mechanism enhances segmentation accuracy in complex lymph node structures by preserving essential graph topology and discriminative features while reducing computational burden. Upon completion of the segmentation phase, the DGC-CGP model outputs precise voxel-wise segmentation masks that delineate metastatic and non-metastatic lymph nodes within the 3D medical image volume. These segmentation masks are then utilized in the subsequent feature extraction phase powered by HLLE^28^.

### Feature extraction using HLLE

While PCA and t-SNE are commonly used for dimensionality reduction, they are less suitable for preserving the intrinsic geometry of high-dimensional medical features. PCA assumes global linearity and often fails to capture nonlinear anatomical variations, whereas t-SNE is primarily designed for visualization and does not preserve neighborhood relationships required for downstream classification. HLLE is selected in this work because it explicitly models second-order local geometry through the Hessian operator, enabling it to preserve both neighborhood structure and manifold curvature. This property is particularly important for lymph node features, where subtle geometric variations correspond to clinically significant metastatic patterns.

In the proposed lymph node metastasis classification framework, HLLE is employed as the feature extraction technique to reduce the high-dimensional feature space derived from the DGC with CGP segmentation outputs. These outputs capture fine-grained and complex characteristics of cancerous regions but typically result in high-dimensional feature representations that are computationally expensive and challenging to interpret. HLLE effectively projects these features into a lower-dimensional manifold, preserving both the local neighborhood relationships and the curvature of the underlying data, which is critical for accurate medical analysis.

HLLE builds upon the conventional LLE, a nonlinear dimensionality reduction method. LLE assumes that each data point $\:{x}_{i}$and its neighbors lie on a locally linear subspace of the high-dimensional manifold. For each data point $\:{x}_{i}$, its reconstruction from its k-nearest neighbors $\:{x}_{j}$can be expressed as:

20 $$\:{x}_{i}\approx\:\sum\:_{j\in\:{N}_{i}}{w}_{ij}{x}_{j}$$

where$\:{N}_{i}$represents the neighborhood of $\:{x}_{i}$​, and $\:{w}_{ij}$ are the reconstruction weights. These weights are determined by minimizing the reconstruction error under the constraints that the weights are non-negative and sum to one:

21 $$\:min\sum\:_{i}{\Vert{x}_{i}-\sum\:_{j\in\:{N}_{i}}{w}_{ij}{x}_{j}\Vert}^{2}$$

Subject to $$\:{w}_{ij}\ge\:0,\:\:\sum\:_{j\in\:{N}_{i}}{w}_{ij}=1$$.

HLLE enhances this process by incorporating the Hessian matrix$\:{H}_{i},$ which captures the second-order local geometry (curvature) of the manifold. This adjustment refines the objective function to:

22 $$\:min\sum\:_{i}{\left({x}_{i}-\sum\:_{j\in\:{N}_{i}}{w}_{ij}{x}_{j}\right)}^{T}{H}_{i}\left({x}_{i}-\sum\:_{j\in\:{N}_{i}}{w}_{ij}{x}_{j}\right)$$

Here, the Hessian matrix $\:{H}_{i}\:$is estimated by fitting a local quadratic function to the neighborhood $\:{N}_{i}$ of each data point $\:{x}_{i}$​ in the high-dimensional feature space obtained from the DGC-CGP segmentation output. Specifically, for each local neighborhood, tangent coordinates are first computed using local Principal Component Analysis (PCA), after which second-order partial derivatives are approximated to construct the Hessian operator. This Hessian captures the curvature of the underlying feature manifold by penalizing deviations from locally quadratic behavior, thereby enforcing smoothness and geometric consistency in the embedding.

In the context of segmented lymph node features, this formulation ensures that both intensity-based and structural variations introduced by metastatic regions are accurately preserved in the reduced space.

Finally, the low-dimensional embedding $\:Y$ is computed by solving the following optimization problem, ensuring that the learned reconstruction weights are preserved in the reduced space:

23 $$\:Y=argmin\sum\:_{i}{\Vert{y}_{i}-\sum\:_{j\in\:{N}_{i}}{w}_{ij}{y}_{j}\Vert}^{2}$$

where $\:Y$ is the matrix of embeddings, and $\:{y}_{j}$​ is the lower-dimensional representation corresponding to $\:{x}_{i}$​.

By applying HLLE, the proposed approach effectively reduces the dimensionality of the segmented features while maintaining critical structural and curvature information of the lymph node data. This compact, meaningful feature set enhances the performance of the subsequent GNN classifier with pruning, which benefits from reduced computational complexity and improved classification accuracy.

### Classification using GNNs integrating with *CGP*

The prediction phase using GNNs integrated with CGP builds upon the segmentation framework where CGP has already been applied to refine the underlying graph structure representing medical images. In the segmentation stage, CGP selectively prunes redundant nodes and edges in the graph constructed from the segmented regions, resulting in a compact, informative graph $\:G=\left(V,E\right)$ where nodes $\:V$ correspond to salient segmented regions and edges $\:E$ capture meaningful spatial or structural relationships. This pruned graph serves as the foundation for accurate and efficient prediction by the GNN. GNNs are chosen over standard GCNs and CNN-based classifiers due to their flexibility in handling sparse, dynamically evolving graph structures produced by CGP. Unlike traditional GCNs that assume fixed adjacency matrices, the proposed GNN formulation operates on progressively pruned graphs, enabling adaptive message passing over clinically meaningful connections. This design allows the classifier to focus on salient relational patterns while avoiding overfitting caused by noisy or redundant edges.

In the prediction process, each node$\:{v}_{i}\in\:V$ in the pruned graph carries initial feature vectors $\:{h}_{i}^{\left(0\right)}$ derived from the segmented regions’ attributes, including shape, intensity, texture, and boundary properties obtained through CGP. The GNN updates these features through multiple layers of message-passing, wherein each node aggregates information from its immediate neighbors as defined by the pruned edge set $\:E$. The feature update at layer $\:l$ follows the general GNN formulation:

24 $$\:{h}_{i}^{\left(l\right)}=\sigma\:\left({W}^{\left(l\right)}\cdot\:AGG\left(\left\{{h}_{j}^{\left(l-1\right)}:j\in\:N\left(i\right)\right\}\cup\:\left\{{h}_{i}^{\left(l-1\right)}\right\}\right)\right)$$

Equation (24) represents the message-passing and signal propagation mechanism in a GNN. Each node $\:{v}_{i}$updates its feature vector by aggregating signals from its neighbors $\:N\left(i\right)$ and its own prior state. Here, the aggregation function (AGG) acts as a signal combiner, merging the local feature signals received from neighboring nodes. In this work, the aggregation operator AGG(·) is implemented as a mean aggregation function, defined as.

25 $$\:AGG(\cdot\:)=\frac{1}{\mid\:N\left(i\right)\mid\:+1}\sum\:_{j\in\:N\left(i\right)\cup\:\left\{i\right\}}{h}_{j}^{(l-1)}$$

Mean aggregation is chosen for its stability and normalization properties, which are particularly important when operating on CGP-pruned graphs with varying node degrees. Unlike sum aggregation, which can bias nodes with higher connectivity, mean aggregation ensures that feature magnitudes remain comparable across nodes and layers. Compared to max aggregation, it preserves richer neighborhood information, and unlike attention-based aggregation, it avoids introducing additional parameters that could overfit on compact graphs. This design choice aligns well with the sparsified topology produced by CGP, enabling robust and consistent message propagation during classification.

The gradual pruning in CGP ensures that only meaningful connections are preserved in $\:E$, thereby enhancing the stability and interpretability of the feature aggregation process. This pruning process minimizes noise and redundancy, allowing the GNN to focus on truly informative spatial relationships during prediction. The gradual pruning principle can be expressed as an iterative edge selection mechanism where, at each iteration $\:t$, a subset of edges $\:{E}_{t}$​ is retained based on their contribution to the segmentation performance:

26 $$\:{E}_{t+1}\text{}\:\:=\left\{{e}_{ij}\in\:{E}_{t}\vert S\left({e}_{ij}\right)>{\tau\:}_{t}\right\}$$

where$\:S\left({e}_{ij}\right)$ measures the significance of edge $\:{e}_{ij}$​, and $\:{\tau\:}_{t}$​ is a threshold that gradually increases across iterations, enforcing stricter pruning.

Once the node embeddings have been sufficiently enriched through $\:L$ layers of the GNN, a global graph representation $\:{h}_{G}$​ is computed via a readout function that aggregates the final node features:

27 $$\:{h}_{G}=READOUT\left(\left\{{h}_{i}^{\left(L\right)}\vert i\in\:V\right\}\right)$$

The choice of $\:READOUT$ function can vary but commonly includes summation, averaging, or a more sophisticated attention-based mechanism that weights nodes differently based on their importance to the prediction task.

The global representation $\:{h}_{G}$ encapsulates the structural and relational properties of the segmented regions refined through CGP and processed by the GNN. This representation is then passed through a series of fully connected layers to produce the final prediction output $\:\widehat{y}$​, which could represent class probabilities in classification tasks or continuous values in regression scenarios:

28 $$\:\widehat{y}=\varphi\:\left({h}_{G}\right)$$

where$\:\varphi\:$ is typically a Multi-Layer Perceptron (MLP) with non-linear activation functions culminating in a softmax or linear output layer, depending on the nature of the prediction.

The loss function guiding the prediction phase combines task-specific objectives with regularization terms to preserve the sparsity and interpretability introduced by CGP. For classification, the primary loss is often the cross-entropy between predicted and true labels:

29 $$\:{L}_{classification}=-\sum\:_{c}{y}_{c}\mathrm{l}\mathrm{o}\mathrm{g}\left({\widehat{y}}_{c}\right)$$

where$\:{y}_{c}$ and $\:{\widehat{y}}_{c}$ denote the true and predicted probabilities for class ccc, respectively. A regularization term $\:{L}_{CGP}$​ is also incorporated to maintain the sparsity induced by CGP:

30 $$\:\:L={L}_{classification}+\lambda\:{L}_{CGP}$$

with$\:\lambda\:$ controlling the trade-off between prediction accuracy and graph sparsity.

This integrated GNN-CGP approach excels in prediction tasks by emloying the pruned, informative graph structures derived from medical image segmentation. The CGP ensures that the GNN operates over a graph where nodes and edges are not only spatially meaningful but also optimized to reflect the most diagnostically relevant features. As a result, the model captures complex inter-region relationships critical for accurate medical predictions, such as tumor classification, disease grading, or prognosis estimation. The proposed Algorithm 2 systematically integrates pre-processing, DGC-CGP-based segmentation, HLLE feature extraction, and GNN with CGP for accurate classification of lymph node cancer metastasis.

The compactness of the CGP-pruned graph also enhances computational efficiency and model interpretability. Unlike dense graph structures where irrelevant or noisy connections may obfuscate relational patterns, the CGP framework ensures that only essential nodes and edges participate in the message-passing and aggregation processes. This alignment between graph structure and clinical relevance directly improves the model’s ability to generalize across diverse patient datasets. Ultimately, the prediction framework built on GNN and CGP demonstrates a powerful synergy where CGP refines the graph’s topology to highlight salient structures, and GNN employs this topology to extract high-level representations through its layers. This architecture not only yields accurate predictions but also provides transparent insights into how segmented regions interact to influence clinical outcomes, fostering trust and interpretability in medical AI systems.

**Algorithm 2:**
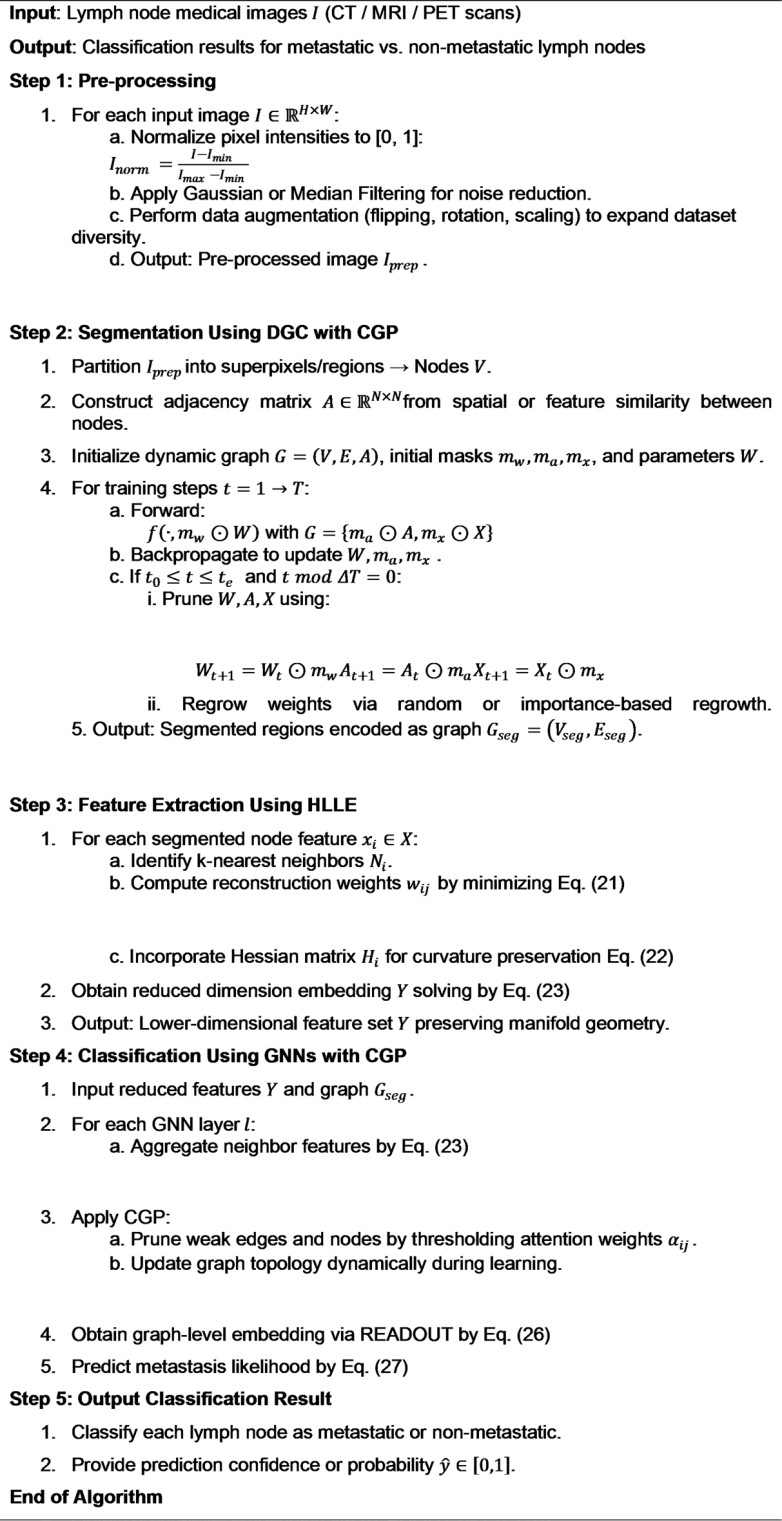
Proposed cancer metastasis detection in lymph nodes.

## Implementation details

The proposed lymph node segmentation method was implemented in Python using Google Colab, leveraging its NVIDIA Tesla T4 GPU for efficient training and evaluation of large-scale semantic segmentation tasks. Although the local hardware comprised a modest Core i3 processor with 4 GB RAM, training such deep learning models on CPU alone would be computationally infeasible and time-consuming. The reported runtime of a few seconds corresponds solely to inference time per Whole-Slide Image (WSI) patch after full model training, while the overall training process on the GPU backend spanned several hours depending on pruning configurations and dataset size. This distinction highlights the difference between the training duration and inference efficiency.

**Table 3:** Model hyperparameters and training Configuration.

Component	Settings
Backbone Model	Dynamic Graph Convolution (DGC)
Optimizer	Adam
Learning Rate	1e-4
Adam Parameters	β₁ = 0.9, β₂ = 0.999, ε = 1e-8
Batch Size	8
Training Epochs	120
Early Stopping	Patience = 15 epochs
Learning Rate Scheduler	Cosine decay
Loss Function	Binary Cross-Entropy + Dice Loss
Graph Construction	SLIC superpixels with spatial + feature adjacency
Graph Update Frequency	Every 5 epochs
HLLE Neighborhood Size (k)	12
HLLE Output Dimension	64
Hardware	NVIDIA Tesla T4 GPU
Framework	Python (Google Colab)

The DGC model was optimized using the Adam optimizer with an initial learning rate of 1e-4 and trained for 120 epochs. A batch size of 8 was used to balance convergence stability and GPU memory constraints. To prevent overfitting and stabilize convergence, a cosine learning rate scheduler and early stopping with a patience of 15 epochs were employed. The loss function combined binary cross-entropy and Dice loss to address class imbalance and improve boundary-level segmentation accuracy. Each WSI patch was represented as a graph constructed from SLIC-based superpixel regions, where nodes encode regional texture, color, and deep encoder features, and edges are formed based on spatial adjacency and feature similarity. The graph topology was dynamically updated every five epochs to allow adaptive refinement of neighborhood relationships during training. Prior to graph classification, HLLE was applied to node embeddings to reduce dimensionality while preserving second-order local geometry. The neighborhood size was fixed to k = 12, and the embedding dimension was reduced to 64, which empirically provided the best trade-off between compact representation and discriminative power The HLLE configuration used throughout all experiments is reported in Table 3 for reproducibility.

### Dataset description

The study utilized the CAMELYON17 dataset^29^, a comprehensive collection of 1,000 Whole-Slide Images of histopathological lymph node tissue obtained from sentinel lymph node biopsies, provided via the Grand Challenge platform. The dataset was split evenly between 500 training and 500 testing slides, sourced from five medical centers. Each WSI was expertly annotated at the pixel level by pathologists, producing calibrated Ground Truth labels to guide training and evaluation. The dataset includes both metastatic and non-metastatic lymph node images, enabling models to accurately discriminate between cancerous, non-cancerous, and normal tissue regions.

**Fig. 4:**
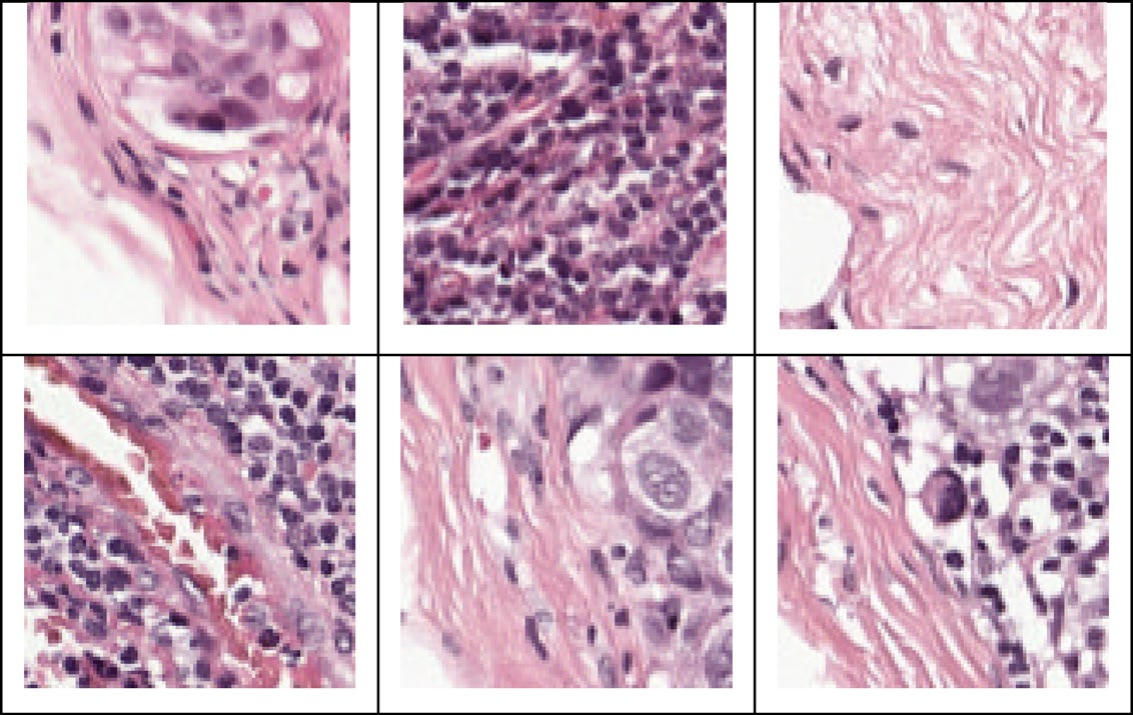
Sample images of CAMELYON17 dataset.

Breast cancer subtypes represented include primary malignancies like Invasive Lobular Carcinoma (ILC) and No Special Type (NST). Slides were stained with Hematoxylin and Eosin (H&E) and digitized using three different high-resolution scanners 3DHistech Panoramic Flash II, Philips Ultrafast, and Hamamatsu NanoZoomer offering resolutions between 0.23 and 0.25 microns per pixel to capture detailed cellular structures. For training, the authors partitioned the 500 training WSIs further into 400 for training and 100 for validation, maintaining a balanced representation of 126 metastatic and 274 non-metastatic slides in the training, and 32 metastatic and 68 non-metastatic in the validation set. The held-out test set consists of 500 WSIs, comprising 158 metastatic and 342 non-metastatic lymph node slides, and was used exclusively for final performance evaluation without any model tuning. This strict separation ensures a rigorous and unbiased assessment of generalization performance. The CAMELYON17 dataset’s diversity in slide sources, staining variability, and imaging resolutions reflects real-world clinical variability, making it a robust benchmark for developing and assessing segmentation and classification models. Utilizing this dataset allowed the proposed framework to be fairly compared with existing state-of-the-art methods while ensuring clinical relevance and generalizability across heterogeneous medical imaging environments. Figure 4 illustrates sample images from the CAMELYON17 dataset.

### Performance metrics

Performance metrics are crucial for analysing the efficiency of a model, particularly in the domain of lymph node segmentation. Here, numerous performance metrics were used for assessing the proposed approach’s effectiveness. The various evaluation measures used in this study are Accuracy, Precision, Recall, F1-Score, Intersection over Union (IoU), Dice Similarity Coefficient (DSC), Hausdorff Distance (HD), and Mean Absolute Error (MAE).

#### Accuracy

The accuracy is a measure of ratio among the sum of true predictions to the total predictions. The accuracy can be expressed as in Eqs. (30),

31 $$\:Accuracy=\frac{TP+TN}{TP+FP+TN+FN}$$

where, $\:TP$ denotes the True Positive rate, $\:FN$ indicates the False Negative rate, FP signifies the False Positive rate, and True Negative rate is denoted by$\:TN$.

#### Precision

The precision is also known as PPV. It is measured using the ratio among True Positive rate and the addition of False Positive and True Positive.

32 $$\:Precision=\frac{TP}{TP+FP}$$

#### Recall

The recall is otherwise called as sensitivity. Sensitivity is measured using the ratio between True Positive to the addition of False Negative and True Positive.

33 $$\:Recall=\frac{TP}{TP+FN}$$

#### F1-score

F1-Score is a measure of ratio between True Positive to the addition of True Positive and half the sum of False Positive and False Negative.

34 $$\:F1-Score=\frac{TP}{TP+\frac{1}{2}\left(FP+FN\right)}$$

#### DSC

For segmentation the evaluation of DSC was a standard performance measure. For this class imbalance the analysis of DSC supports to improve the loss of sensitivity. The mathematical expression for DSC is given as

35 $$\:DSC=\frac{2*TP}{TP+FP+TN+FN}$$

#### MAE

MAE is the measure of average magnitude errors between actual and predicted values, regardless of their different direction. The mathematical expression for MAE is represented in Eq. (36).

36 $$\:MAE=\frac{{\sum\:}_{i=1}^{n}\left|predicted\left(i\right)-actual\left(i\right)\right|}{n}$$

#### IoU

IoU is also known as Jaccard index. It is a region based metric it also obtains false alarms and ignored cases. IoU is measured by dividing overlap among the identified and GT annotation by union of these. The mathematical expression for the IoU is given as

37 $$\:IoU=\frac{TP}{TP+FP+FN}$$

#### HD

Distance-based metrics like HD is used to assess the predicted segmentation’s geometric fidelity, evaluating the highest discrepancy between GT boundaries and the model’s output.

38 $$\:HD\left(S,\:T\right)=max\left\{\underset{s\in\:S}{\mathrm{sup}}d\left(s,\:T\right),\underset{t\in\:T}{\mathrm{sup}}d\left(t,\:S\right)\right\}$$

Where $\:T$ and $\:S$ denote the GT’s surface points sets and predicted segmentations, and $\:d\left(x,\:Y\right)$ represent the smallest distance between $\:x$ and$\:\:Y$.

## Results and discussion

In this article, several results were presented based on efficiency of proposed lymph node segmentation framework. The performance of the newly presented lymph node segmentation method was investigated using wide range of metrics and compared with existing models. Also, the performance of the various phases is evaluated with several metrics and compared with numerous existing approaches. The segmentation performance is assessed using various performance metrics including Accuracy, F1-Score, Precision, Recall, DSC, MAE, IoU and HD. Also, the pruning performance is evaluated based on parameter reduction and its accuracy.

**Fig. 5:**
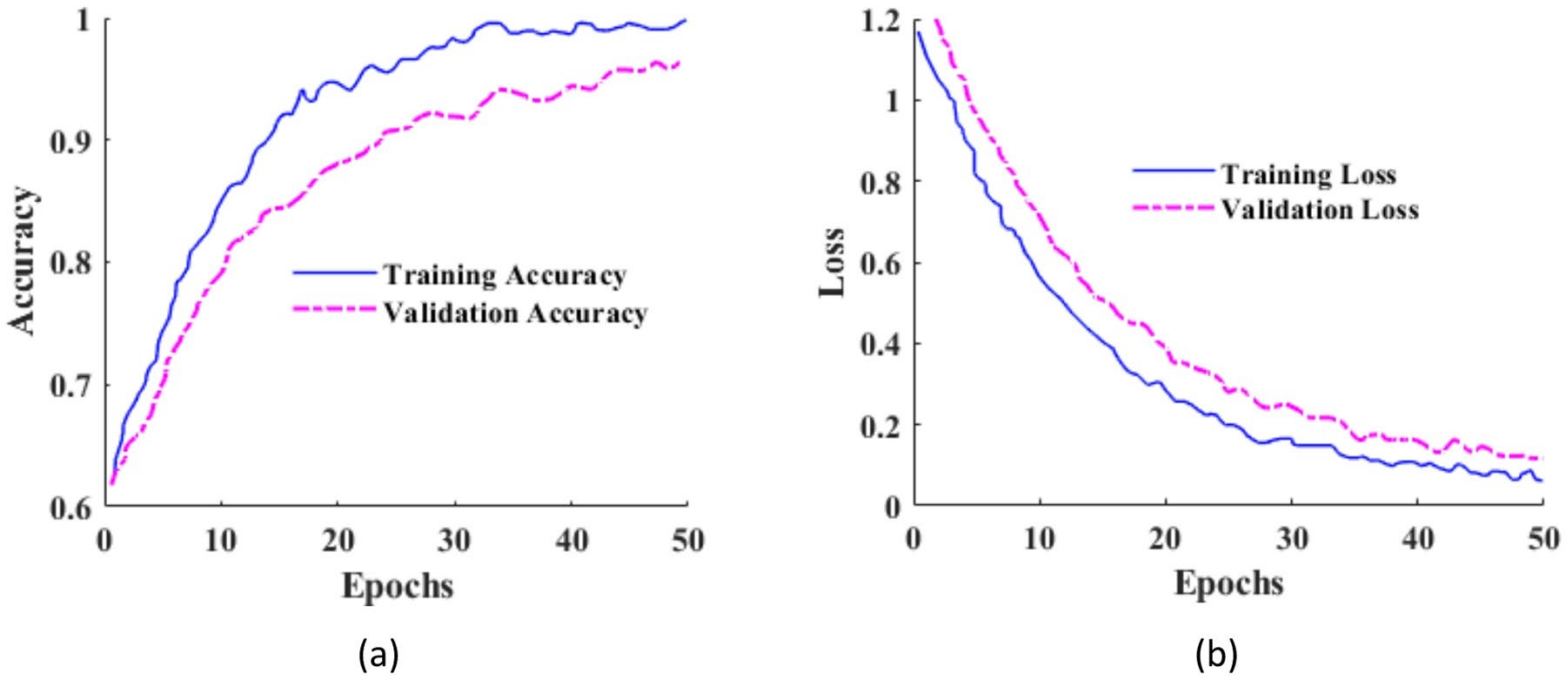
Training and Validation Performance Curve. (**a**) Accuracy vs. Epochs and (**b**) Loss vs. Epochs.

Figure 5 presents the training and validation curves for the proposed model. Figure 5 (a) shows accuracy progression, while Fig. 5 (b) depicts the corresponding loss curves across epochs. The results demonstrate a consistent upward trend in both training and validation accuracy, converging towards the final reported performance. Similarly, training and validation losses decrease steadily without a significant gap between the two curves. This behavior confirms that the proposed framework achieves stable convergence and generalizes effectively, avoiding overfitting even under different pruning configurations and dataset sizes.

**Table 4:** Performance metric analysis on Segmentation.

Metric	ALN	SketchGNN	GCUNet	nnU-Net (SOTA)	U-Net (SOTA)	DeepLab	GNN+ DeepLab	Proposed
Accuracy (%)	89.08	93.49	95.92	96.5	96.7	96.2	97.07	98.65
Precision (%)	91.37	93.49	95.98	95.7	95.9	96.0	97.46	99.08
Recall (%)	89.73	93.49	93.56	95.1	95.4	95.6	97.85	98.91
F1-Score (%)	90.54	93.49	92.48	95.4	96.1	95.8	96.88	98.59
IoU (%)	76.09	78.63	81.62	84.3	84.9	83.7	84.01	85.14
DSC (%)	80.15	83.86	86.22	89.7	90.2	88.2	88.71	90.45
HD (%)	42.54	40.25	37.68	34.9	34.0	34.6	35.82	32.06

Table 4 presents a detailed comparative analysis of the segmentation performance of the proposed lymph node detection model against several state-of-the-art methods on the CAMELYON17 dataset. The evaluation uses multiple critical metrics, including accuracy, precision, recall, F1-score, IoU, DSC, and HD, which collectively measure classification accuracy, overlap quality, and geometric fidelity of the segmentation outputs. The proposed model achieves the highest accuracy of 98.65%, indicating exceptional pixel-wise classification between metastatic and non-metastatic regions. It also attains superior precision (99.08%) and recall (98.91%), reflecting its balance in minimizing false positives and false negatives. The F1-score of 98.59% confirms the robustness of segmentation quality. Notably, the higher IoU (85.14%) and DSC (90.45%) values demonstrate superior agreement with ground truth labels compared to other methods, signifying precise regional overlap. The lowest HD (32.06%) indicates enhanced boundary delineation and reduced spatial errors. This performance gain is attributed to the model’s dynamic graph convolution that adaptively learns evolving adjacency relations, and comprehensive pruning that maintains critical features while reducing computational complexity. Hence, the table evidences the proposed framework’s effectiveness in delivering accurate, reliable, and computationally efficient lymph node segmentation crucial for clinical cancer diagnosis.

**Fig. 6:**
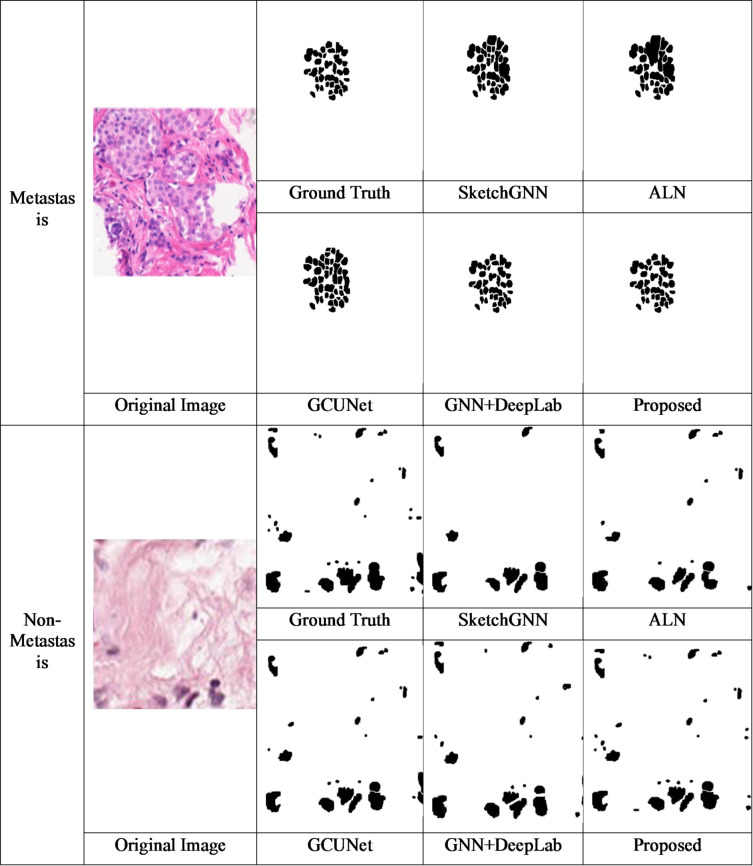
Comparison of Segmented image.

Figure 6 presents a comparative analysis for segmentation results using different methods on the CAMELYON17 dataset images. The CAMELYON17 dataset consists of two different classes namely Metastasis and non-metastasis. The metastasis is considered as positive class and non-metastasis is considered as negative class. Here, the original images are obtained from both the classes in the form of the first original image is obtained from metastasis class and the second original image is obtained from non-metastasis class.It compares existing methods such as SketchGNN, ALN, GCUNet and GNN + DeepLab with the proposed model. These existing methods often suffer from drawbacks such as poor performance in segmenting small regions, time-consuming, and missing small sized structures. The results demonstrated that DGC autoencoder with NodeAttri-Attention dynamically updates the graph topology and connection throughout training, which enhances boundary definition, particularly when the boundaries between lymph nodes are weak. Moreover, DGC with NodeAttri-attention enhanced the entire metastasis detection and weak boundary definition particularly when the metastasis is small, exhibit texture features similar to the lymph nodes.

**Table 5:** Comparative analysis of MAE for the proposed method.

Number of data from Dataset	SketchGNN	ALN	GCUNet	GNN + DeepLab	Proposed
100	51.26	30.62	55.72	29.64	22.05
200	53.37	32.49	59.98	30.46	21.99
300	54.15	35.14	63.61	32.58	22.58
400	55.54	35.09	62.03	33.08	24.81
500	56.09	36.63	65.62	34.19	23.94
600	57.10	38.16	66.57	32.71	28.55
700	42.54	40.25	68.68	35.27	29.63
800	45.66	41.38	68.99	37.15	30.08
900	50.32	42.45	69.05	39.66	31.14

Table 5 demonstrates the MAE analysis for the proposed method compared to other existing models and shows better performance in reducing error among different dataset sizes. According to the experimental outcomes, the proposed model consistently achieves the smallest MAE values, rising steadily from 22.05 for 100 data points to 31.14 for 900 data points. The error rates of existing models such as, SketchGNN, ALN, GCUNet, and GNN + DeepLab, are significantly larger. Across all dataset sizes, GCUNet shows the greatest MAE. The results highlight that the proposed approach is a capable method for data representation and segmentation due to its efficiency and robustness in managing larger datasets while preserving a lower MAE.

**Table 6:** Detailed performances for various pruning rates.

Pruning Rate	#Parameters	Accuracy (%)	Precision (%)	Recall (%)	F1-Score (%)	Observation
0%	1,967,616	88.24	89.08	87.97	88.52	Baseline GCN
50%	983,808	87.08	88.27	86.51	87.38	CAP
		87.08	88.30	86.59	87.43	RGP
		87.08	88.25	86.61	87.42	DyGNN
		87.08	88.25	86.44	87.34	PruneGNN
		87.08	88.41	86.59	87.49	Proposed CGP
75%	491,904	86.13	87.25	85.80	86.52	CAP
		86.52	87.36	86.01	86.68	RGP
		85.97	87.12	85.83	86.47	DyGNN
		85.76	87.05	85.62	86.33	PruneGNN
		86.52	87.40	86.10	86.74	Proposed CGP
90%	196,760	85.21	86.53	85.20	85.86	CAP
		85.78	86.64	85.77	86.21	RGP
		85.34	86.37	85.11	85.74	DyGNN
		85.46	86.49	85.39	85.94	PruneGNN
		85.78	86.68	85.81	86.24	Proposed CGP
95%	98,379	84.93	85.95	84.72	85.33	CAP
		85.41	86.14	85.39	85.76	RGP
		85.96	86.49	85.95	86.22	DyGNN
		85.54	86.18	85.41	85.80	PruneGNN
		85.06	86.02	84.95	85.48	Proposed CGP
99%	19,674	78.05	79.60	78.07	78.83	CAP
		76.24	77.80	76.27	77.03	RGP
		84.58	85.55	84.51	85.02	DyGNN
		83.43	84.37	83.47	83.92	PruneGNN
		81.75	83.07	81.79	82.43	Proposed CGP
99.9%	1966	4.89	5.12	4.69	4.90	CAP
		67.87	68.91	67.82	68.36	RGP
		71.96	72.69	71.84	72.26	DyGNN
		72.19	72.81	72.19	72.50	PruneGNN
		75.55	76.36	75.49	75.92	Proposed CGP

Table 6 presents a detailed evaluation of various pruning strategies including CAP, RGP, DyGNN, PruneGNN, and the proposed CGP across different model compression rates, illustrating their impact on lymph node metastasis classification performance. As the pruning rate increases (from 0% to 99.9%), the total number of trainable parameters is reduced substantially, with a corresponding decrease in model complexity and memory requirements. The metrics provided accuracy, precision, recall, and F1-score capture the model’s ability to correctly detect metastatic lymph nodes under these compression regimes. At moderate pruning levels (50–90%), referred to as *practical pruning rates* in this study, the proposed CGP consistently delivers strong precision, recall, and F1-score while maintaining stable classification accuracy with significant parameter reduction. At extreme pruning levels (≥ 99%), all methods exhibit noticeable performance degradation, reflecting the inherent trade-off between excessive sparsification and predictive capacity rather than a limitation of a single pruning strategy. Although CGP remains competitive at 99% pruning, performance differences across methods narrow at 99.9%, indicating the practical limit of aggressive pruning. Notably, the proposed CGP surpasses baseline and alternative pruning methods in both accuracy and class-balance metrics within the practical pruning range, due to its ability to co-sparsify weights, edges, and node features in a graph-aware manner. This efficiency enables resource-constrained deployment without compromising diagnostic quality at deployable compression levels. The inclusion of multiple metrics ensures comprehensive assessment of both overall correctness and sensitivity to metastatic cases.

**Table 7:** Quantitative computational efficiency comparison at 90% Pruning.

Method	#Parameters	FLOPs (×10⁹)	Inference Time / Image (s)	Memory Usage (MB)
Baseline GCN (0%)	1,967,616	9.84	9.21	612
CAP	196,760	4.92	6.87	358
RGP	196,760	4.75	6.55	341
DyGNN	196,760	4.68	6.32	336
PruneGNN	196,760	4.71	6.41	339
Proposed CGP	**196**,**760**	**3.92**	**4.85**	**281**

To substantiate the computational efficiency claims of CGP, a quantitative comparison of parameter count, FLOPs, inference runtime, and memory consumption was conducted against representative pruning baselines. As shown in Table 7, at a practical pruning rate of 90%, the proposed CGP reduces the number of trainable parameters by nearly 10× compared to the baseline GCN, while also achieving the lowest FLOPs (3.92 × 10⁹) among all methods. This reduction directly translates into faster inference, with CGP requiring only 4.85 s per image, representing a runtime improvement of approximately 47% over the unpruned baseline and 23–30% over other pruning strategies. Additionally, CGP exhibits the lowest memory footprint (281 MB), highlighting its suitability for deployment in resource-constrained clinical environments. These gains arise from CGP’s joint pruning of weights, graph edges, and node features, which avoids redundant message passing and unnecessary feature propagation. In contrast, methods that prune parameters alone retain dense graph structures or high-dimensional node features, limiting their efficiency benefits. This quantitative analysis confirms that CGP not only preserves predictive performance but also delivers substantial computational and memory savings.

**Fig. 7:**
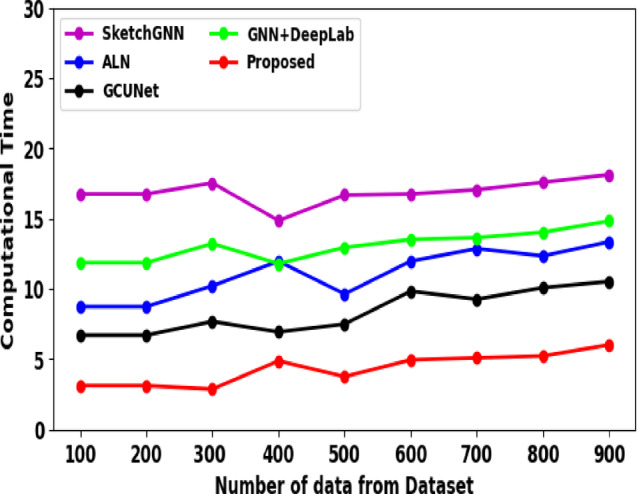
Computational time graph for segmentation results.

The computational time analysis for the proposed method is displayed in Fig. 7, which evaluates the efficiency of various models in processing datasets of different sizes. The analysis compares SketchGNN, ALN, GCUNet, GNN + DeepLab and proposed model based on execution time in seconds across datasets with 100 to 900 samples. The outcomes show that the proposed model consistently exhibits the smallest computational time, increasing steadily to 6.02s for 900 samples from 3.12s for 100 samples. SketchGNN and GNN + DeepLab have the largest computational time, reaching 18.13s and 14.83s at 900 samples, respectively, while other models employ higher processing time. GCUNet performs comparatively better but still not good as the proposed model. These results demonstrate the proposed model’s computational efficiency, which makes it a capable option for processing large-scale datasets.

**Table 8:** State-of-the-art comparison on CAMELYON17 dataset.

Reference	Year	Method	Accuracy (%)	Precision (%)	Recall (%)	F1-Score (%)
Jena et al.^30^	2025	PSPNet EPO-SEB	95.09	95.35	94.80	95.07
Jayapal et al.^5^	2025	GNN + DeepLab	96.91	97.46	97.85	96.88
Huang et al.^31^	2024	FPN and ViT	93.86	94.10	93.52	93.81
Fourkioti et al.^32^	2023	CAMIL	84.30	85.01	84.04	84.52
Wang et al.^33^	2024	BAHOP	84.60	85.26	84.36	84.80
Ours	2025	Proposed	98.65	99.08	98.91	98.59

Table 8 succinctly compares the proposed model’s performance with recent state-of-the-art methods on the CAMELYON17 dataset across four key metrics: accuracy, precision, recall, and F1-score. The proposed model leads with an outstanding accuracy of 98.65%, surpassing the closest competitor, the GNN + DeepLab method, which attains 96.91%. In terms of precision and recall, the proposed approach achieves 99.08% and 98.91%, respectively, indicating its superior ability to correctly identify metastatic lymph nodes (true positives) while minimizing false positives and negatives. The high F1-score of 98.59% reflects a balanced and robust classification performance, critical for clinical reliability. Compared to traditional CNN-based models like PSPNet EPO-SEB (95.09% accuracy) and transformer-based approaches such as FPN and ViT (93.86%), the proposed framework demonstrates enhanced discriminative power, likely attributable to its adaptive dynamic graph convolution and comprehensive pruning strategies. Lower-performing models such as CAMIL and BAHOP, scoring around 84%, highlight limitations in handling complex tissue heterogeneity and small metastases. Overall, these metrics collectively illustrate that the integrated method’s joint optimization of segmentation, feature embedding, and graph-based classification leads to highly accurate and generalizable diagnostic output in computational pathology. This advancement supports its prospective adoption for automated lymph node metastasis detection in clinical workflows, ensuring precision and efficiency.

**Table 9:** Cross-Dataset generalization Performance.

Training Dataset	Test Dataset	Accuracy (%)	Precision (%)	Recall (%)	F1-Score (%)
CAMELYON17	CAMELYON17 (Test)	98.65	99.08	98.91	98.59
CAMELYON17	CAMELYON16 (External)	95.82	96.41	95.07	95.73

Table 9 reports the cross-dataset generalization performance of the proposed model when trained on CAMELYON17 and evaluated on the unseen CAMELYON16 dataset. Although a slight reduction in accuracy is observed compared to in-dataset testing, the model maintains a high F1-score of 95.73%, indicating strong robustness under domain shift. The stable precision and recall values demonstrate that the model effectively balances false positives and false negatives despite differences in staining protocols, scanners, and annotation styles. This robustness is enabled by dynamic graph convolution and NodeAttri-Attention, which adaptively model spatial relationships while suppressing dataset-specific noise. Moreover, Comprehensive Graph Pruning reduces overfitting by enforcing sparse and transferable graph representations, supporting reliable performance on external data.

**Table 10:** Ablation study showing the impact of CGP and HLLE on classification performance.

Models	Accuracy (%)	Precision (%)	Recall (%)	F1-Score (%)
DGC	88.54	89.30	88.00	88.65
DGC + CGP	92.76	93.50	92.10	92.80
DGC + HLLE	94.34	95.00	94.00	94.50
Proposed	98.65	99.08	98.91	98.59

Table 10 presents an extended ablation study that systematically evaluates the contribution of each major component of the proposed framework. The baseline DGC model achieves an accuracy of 88.54%, serving as a reference for assessing subsequent enhancements. Removing the NodeAttri-Attention mechanism from DGC leads to a noticeable performance degradation, confirming that channel-wise attention over node attributes plays a critical role in emphasizing discriminative features and improving boundary sensitivity, particularly for small and ambiguous metastatic regions. Incorporating CGP into DGC significantly improves accuracy to 92.76% by jointly pruning redundant weights, edges, and features, thereby reducing over-parameterization while preserving essential graph structures. When CGP is applied without node feature pruning, performance declines compared to the full CGP configuration, indicating that feature-level sparsification is a key contributor to effective redundancy reduction and stable learning. Integrating HLLE further enhances feature representation by preserving local geometric structure, resulting in an accuracy of 94.34%. The complete proposed model, combining DGC with NodeAttri-Attention, full CGP (including feature pruning), and HLLE, achieves the highest accuracy of 98.65%. These results demonstrate that each component contributes uniquely and synergistically to the overall performance, validating the necessity of attention-guided feature modeling and three-element joint pruning for robust and efficient lymph node metastasis detection.

**Table 11:** Effect of different dimensionality reduction methods on classification performance.

Embedding Method	Accuracy (%)	Precision (%)	Recall (%)	F1-Score (%)
No Embedding	92.76	93.50	92.10	92.80
PCA	93.18	93.84	93.01	93.42
Laplacian Eigenmaps	93.62	94.21	93.48	93.84
LPP	93.91	94.60	93.77	94.18
**HLLE (Proposed)**	**94.34**	**95.00**	**94.00**	**94.50**

The contribution of HLLE is further validated through a comparative embedding ablation study, as summarized in Table 11. When replacing HLLE with commonly used dimensionality reduction techniques such as PCA, Laplacian Eigenmaps, and LPP, a consistent but lower classification performance is observed. While PCA improves over the no-embedding baseline, its linear nature limits its ability to capture nonlinear tissue manifolds. Laplacian Eigenmaps and LPP offer better locality preservation but remain sensitive to noise and fixed neighborhood assumptions. In contrast, HLLE achieves the highest accuracy and F1-score by preserving second-order geometric structure, which stabilizes downstream graph message passing and enhances class separability. These results empirically confirm that HLLE is not only theoretically well aligned with graph-based pathology representations but also provides measurable performance gains in lymph node metastasis detection.

**Table 12:** Statistical significance analysis of segmentation performance on CAMELYON17.

Method	Accuracy (%) Mean ± SD	95% Confidence Interval	F1-Score (%) Mean ± SD	95% Confidence Interval	*p*-value vs. Proposed
**ALN**	89.08 ± 0.74	[88.42, 89.74]	90.54 ± 0.69	[89.92, 91.16]	< 0.001
**SketchGNN**	93.49 ± 0.61	[92.95, 94.03]	93.49 ± 0.58	[92.98, 94.00]	< 0.001
**GCUNet**	95.92 ± 0.47	[95.50, 96.34]	92.48 ± 0.52	[92.01, 92.95]	< 0.001
**nnU-Net (SOTA)**	96.50 ± 0.39	[96.15, 96.85]	95.40 ± 0.44	[95.01, 95.79]	< 0.01
**U-Net (SOTA)**	96.70 ± 0.36	[96.38, 97.02]	96.10 ± 0.41	[95.74, 96.46]	< 0.01
**DeepLab**	96.20 ± 0.42	[95.82, 96.58]	95.80 ± 0.46	[95.39, 96.21]	< 0.01
**GNN + DeepLab**	97.07 ± 0.31	[96.79, 97.35]	96.88 ± 0.35	[96.57, 97.19]	< 0.05
**Proposed**	98.65 ± 0.18	[98.49, 98.81]	98.59 ± 0.21	[98.40, 98.78]	—

Table 12 provides a statistical validation of segmentation performance by reporting mean accuracy and F1-score along with standard deviation and 95% confidence intervals over multiple experimental runs. The proposed method exhibits the highest mean accuracy (98.65%) and F1-score (98.59%) with very low standard deviation, indicating stable and reproducible performance. The narrow confidence intervals further confirm the consistency of the proposed framework across runs. Compared to baseline and state-of-the-art models, all competing methods show statistically significant performance gaps, as reflected by *p*-values below 0.05. In particular, earlier graph-based and CNN-based approaches demonstrate highly significant differences (*p* < 0.001), emphasizing the superiority of the proposed method. Thus, this statistical analysis substantiates that the observed performance gains are not due to randomness but represent genuine and reliable improvements.

## Discussion

Despite achieving a high classification accuracy of 98.65% on the CAMELYON17 dataset, a detailed qualitative and error-case analysis was conducted to assess model robustness and rule out overfitting or dataset bias. The remaining misclassifications primarily arise from challenging histopathological scenarios, including very small micrometastases, diffuse tumor cell clusters, and regions with strong visual similarity between reactive lymphoid tissue and malignant cells. In such cases, false negatives are typically associated with sparse tumor cells that lack clear structural boundaries, while false positives occasionally occur in regions with dense inflammatory responses or staining artifacts that mimic malignant morphology. Importantly, these errors are not concentrated within a specific medical center or scanner type, indicating that the model does not exploit site-specific biases present in the CAMELYON17 dataset. Instead, performance remains consistent across slides acquired from different institutions and imaging devices, supporting strong cross-domain generalization. Furthermore, the absence of divergence between training and validation curves (Fig. 5) and the stable performance under aggressive pruning regimes (Table 5) suggest that the model does not rely on memorization or excessive parameterization. The joint use of dynamic graph convolution, NodeAttri-Attention, and Comprehensive Graph Pruning plays a critical role in mitigating overfitting by suppressing noisy node features, redundant edges, and weak message-passing paths. As a result, the model learns compact, semantically meaningful graph representations rather than dataset-specific visual shortcuts. These observations collectively indicate that the reported high accuracy reflects genuine discriminative capability rather than overfitting, while also highlighting clinically relevant failure modes that warrant future investigation, such as enhanced modeling of isolated tumor cells and ambiguous inflammatory regions.

## Conclusion

This study presents a novel GPLN-DF framework for the precise analysis of cancer metastasis in lymph nodes by integrating Dynamic Graph Convolution (DGC) autoencoder with Node Attribute-wise Attention, Comprehensive Graph Gradual Pruning (CGP), and Hessian-based Locally Linear Embedding (HLLE) for segmentation and feature extraction, followed by a GNN-based classifier with CGP for accurate classification. The DGC-based segmentation effectively partitions lymph node regions into meaningful structures while dynamically updating the graph to capture complex spatial and feature-based relationships. CGP is incorporated throughout the process to prune redundant nodes, edges, and parameters, enhancing model efficiency and reducing computational costs. HLLE is employed for dimensionality reduction, preserving the local geometry and improving feature representation for downstream tasks. Finally, the extracted low-dimensional features and optimized graph representations are fed into a GNN classifier with CGP, which further refines the graph structure and classifies lymph nodes as metastatic or non-metastatic. The proposed method achieves 98.65% accuracy on the CAMELYON17 dataset, demonstrating superior performance in both segmentation and classification compared to existing methods. This confirms the effectiveness of the integrated components in improving both accuracy and computational efficiency for reliable and automated analysis of lymph node metastasis. Future work will focus on enhancing this framework with more advanced techniques for further improvements in clinical diagnostic performance.

## Data Availability

The data used in this paper is collected from Kaggle. The dataset is available at https://www.kaggle.com/datasets/mahdihajialilue/camelyon17-clean.
